# Spirit of the Foreign Periodicals, &c.

**Published:** 1843-10-01

**Authors:** 


### Spirit of the Foreign Periodicals.

### Pathology of Phlegmasia Dolens—Discussion at the Royal Academy—Remarks.

At a recent *seance* of this learned body, M. *Capuron* read an elaborate report on a memoir by M. *Drousard* on the disease known by the name of Phlegmasia Alba Dolens, and in which the author has attempted to prove that it is generally, if not always, connected with an inflammation of the crural and other veins of the affected limb. The question, it will be seen, gave rise to much discussion and difference of opinion; some assenting to this view of the subject, and others expressing their decided dissent. The result of the whole seems to be, that there are different forms of the disease, and that these are most probably owing to the operation of different causes.

M. *Breschet* objected to the opinion, expressed in the report just read, that *phlegmasia alba dolens* is in all cases the result of an inflammation of the veins in the affected parts. According to his experience, the lymphatic vessels are usually as much implicated in the morbid process as the veins. The phenomena of genuine phlegmasia are certainly not the same as those of ordinary phlebitis; and this, among other reasons, he considered a strong argument against the opinion of M. *Capuron*.

M. *Capuron*, in reply, said that he had by no means denied that the lymphatic vessels may be inflamed in this disease; but only that they are not primarily and necessarily so.

M. *Blandin*:—"It has been long imagined that Phlegmasia alba is a disease peculiar to women. This is not strictly correct; for, although of much more frequent occurrence in females, it is certainly not peculiar to them. When the disease was first recognised and described, it seems to have been regarded as a mere form of œdema; but, in proportion as its phenomena and cause were more attentively studied, it was found that in some cases the veins, in others the lymphatic vessels, and in others still the nerves were in a more or less decided state of inflammation at the time:—this M. *Dance* shewed in his interesting memoir on the subject. Subsequently however it has been discovered that the inflammation of the veins and that of the nerves are not of a necessary, but only of an occasional, coincidence in phlegmasia; and that the only essential pathological character of the disease is an inflammation of the absorbent vessels of the limb. We must admit, however, that the inflammation of the veins sometimes precedes that of the absorbents, and we are therefore obliged to dissent from the opinion of our colleague, M. *Breschet*, who seems to regard the last-named vessels as invariably the seat of the disease in question."

M. *Velpeau*.—"Twenty years ago, when I published my first observations on this disease, it was almost universally maintained that the pathological cause was an inflammation of the lymphatic vessels of the limb. In consequence of several post-mortem examinations, I felt convinced that the veins were often more or less affected, and I expressed this opinion publicly. About the same time, Dr. *Davis* came to nearly the same conclusions in England. I have had very many opportunities, since that time, of studying the diseases of the venous and lymphatic systems, and have treated not a few cases of phlegmasia alba, and I now feel assured of the truth of the following two propositions: *first*, that the opinion of M. *Dance* and others, as to the nerves being in some cases inflamed, is utterly erroneous; and *secondly*, that the disease is primarily seated in the lymphatic vessels of the thigh; that a phlebitis is not necessarily existent; and, when it is, that it is only of consecutive or secondary development. But while I say this, I must also distinctly state that, in almost all *post-mortem* examinations of the affected parts, both sets of vessels have been found more or less seriously diseased. The phenomena of the disease in its first stage tend much to shew that it is rather an *angeioleucitis* than a *phlebitis* that we have to deal with. There is then a general engorgement of the limb, with patches here and there of diffused redness, and irregular uneven nuclei or nodules of tumefaction, which are not necessarily found along the *trajet* of the veins, as in genuine phlebitis. The constitutional symptoms too, in proper phlegmasia dolens, are certainly not the same as in phlebitis; and it is only when both sets of vessels happen to be simultaneously implicated that we ever meet with the symptoms which denote the occurrence of purulent resorption and infection. In conclusion, I will briefly repeat my opinion that genuine Phlegmasia alba dolens is attributable to an angeioleucitis or inflammation of the lymphatic vessels, accompanied with an inflammation of the adjacent cellular tissue, and occasionally also with inflammation of one or more of the veins of the limb."

M. *Capuron* here reminded the Academy that the opinion now expressed by the preceding speaker differs in almost every essential particular from that which he expressed not long ago, and pointed out the striking contradiction between M. Velpeau's present sentiments and those which he has published in his writings.

M. *Cloquet*.—"I agree with M. *Blandin* in the sentiment which he has expressed in his remarks, that phlegmasia alba is not necessarily or invariably met with in the female sex; as cases of it have unquestionably occurred, in my own practice, among youths and men. I have experienced no little difficulty in forming any exact or definite opinion as to the ætiology of this disease. If I were to trust exclusively to the results of the post-mortem examinations which I have made, I should indeed be utterly perplexed what to say; but, by having carefully watched the progress of many cases from their first stage, I have been enabled to form clearer and more accurate notions, as I think, on the subject under consideration. The result of my observations is that, as a general remark, the cellular tissue is primarily the seat of the œdematous swelling; that an inflammatory engorgement, accompanied with effusion, takes place at first; and that it is only consecutively that either the lymphatic vessels or the veins become affected. In my opinion we may regard Phlegmasia alba as a specific exhalant inflammation of the cellular tissue, with or without an accompanying inflammation of the veins or lymphatic vessels of the part."

M. *Moreau* (the eminent obstetrical physician) took nearly the same view of the ætiology of phlegmasia alba as M. *Cloquet*. In his opinion, it is a specific disease which should not be confounded with an inflammation either of the absorbents or of the veins. "Phlebitis is always a serious, and often a most dangerous, disease; phlegmasia alba, when uncomplicated with other lesion, is comparatively mild and innocuous. In proof that there is something special about the disease, we have only to bear in mind the circumstances which usually give rise to it. Is it not remarkable that often it does not occur for 15, 18, or 20 days after delivery, when the usual exciting causes of suppuration have entirely passed away? Does not this circumstance alone suffice to shew that the disease depends most frequently on the neglect of certain precautions on the part of women who have been recently delivered? According to my own experience, it is generally owing to checked perspiration under such circumstances. At first there seems to be nothing but an indolent swelling, a simple engorgement; this, if not relieved, is apt to be followed by inflammation of the absorbents, and in some cases of the veins also. It is only when the last-named lesion is present, that there is any cause for alarm as to the issue of the case. I should define the disease in its primary stage to be 'une veritable inflammation exhalante du tissu cellulaire.'"

M. *Berard:* "I have listened with a great deal of pleasure to the remarks which have just been made by MM. *Cloquet* and *Moreau;* for I must frankly confess that I have never very clearly understood what part the alleged inflammation of the absorbents and veins has been supposed to act in the history of phlegmasia dolens. When the superficial absorbents of an extremity are really inflamed, the phenomena are usually sufficiently obvious to prevent any mistake in the diagnosis; and certainly these phenomena are not those generally exhibited by the disease in question.

With crural phlebitis, it might be more easy to reconcile the symptoms; for M. *Bouillaud* has shewn that an adhesive inflammation of a vein is apt to be followed by œdematous swelling of the part and its neighbourhood, in consequence of the obstruction to the free return of the blood. But, notwithstanding this plausible explanation, I must confess that I am much more inclined to adopt the pathological views of MM. *Cloquet* and *Moreau.* There is a fact which, although not bearing directly on the subject under consideration, may not be undeserving of notice here, I allude to the tendency which exists in puerperal women to the formation of diffused abscesses."

M. *Andral*: "The expression, Phlegmasia alba dolens, is a very unfortunate one, and has tended not a little to keep up the obscurity that hangs over the subject: it is a complex term that embraces a variety of morbid alterations dissimilar from each other, and thus tends to perpetuate confusion. I agree with those gentlemen who have asserted that the disease is not confined, (as alleged by M. *Rochoux,*) to the female sex. I have seen unquestionable cases of it among men; but in them, as well as in women, always associated with a lesion of some of the pelvic viscera. I feel satisfied of the truth of this position, that, wherever there is a phlegmasia dolens, we may at once suspect that there is something wrong within the pelvis. The 'point de depart' of the disease is an obstruction of the chief vein of the limb; this is the cause of the œdema.

"In every case which I have examined by dissection, I have found almost uniformly a correspondence between the lesion of the veins and some inflammatory process within the pelvis—the diagnosis of which, it must be confessed, is often exceedingly difficult during life.

"But I do not mean to assert that this complex disease is always, although certainly generally, dependent upon a mere obliterative phlebitis. Besides the lesion of the veins, there may be a lesion of the absorbent vessels, and also of the cellular tissue. In women the disease is generally a result of delivery; in men, either of some morbid affection within the pelvis, or of an injury of the lower parts of the limb."

M. *Gerardin* expressed his surprise that none of the preceding speakers had alluded to the connexion that certainly exists between the occurrence of the disease, at least in puerperal women, and the state of the mammary secretion. It is acknowledged by all writers, that it is more frequent in those who do not suckle their children than in those who do; and the sudden suppression of the milk has often been known to be quickly followed by an attack of phlegmasia dolens, as well as by sudden effusions into the cavity of the chest, abdomen, or of some of the joints.

Now the diseased action, in both sets of cases, is nearly the same; in the one, the effusion takes place into a cavity, while in the other, it is into the subcutaneous cellular tissue. It is sometimes surprising to see with what rapidity such effusions are induced; their gradual increase may be watched from one hour to another. It is usually asserted that Phlegmasia alba is of much more frequent occurrence in the lower than in the upper extremities. Now the very reverse seems to me to be the case. (We do not remember having ever met with any statement to this effect before.) It has also been said that the disease is seldom attended with any danger; but such an opinion must be received with due caution, as a sudden effusion may take place into one of the internal cavities, or into the sheaths of the blood-vessels, and prove fatal either rapidly, or by a slow wasting process.—*Gazette Medicale.*

*Remarks.*—The attentive reader cannot fail to have remarked the great and more than usual discrepancy of opinion among a set of eminent men, the members of one of the first medical societies of the age, as to the nature of a disease, which is not of very unfrequent occurrence. We are told by one learned academician that the veins are the parts most affected; by another, that the lymphatic vessels are usually the seat of the morbid action; by a third, that both sets of vessels, and the nerves of the limb also, are always more or less inflamed; and by a fourth, that the disease is quite independent of all these parts, and is truly and essentially one of the cellular tissue. May we not, *à priori,* suspect from this very discordance of opinion, that the truth really lies, not in one nor in another of these doctrines, but in the *ensemble* of all put together; and that the disease in question does not uniformly or invariably commence in one part alone? Perhaps it might be said with more accuracy that, under the name of phlegmasia dolens, different diseases, more or less dissimilar from each other, are grouped together as if they were all cognate affections.

For our own part, we are certainly inclined to think so, and we cannot but express our surprise that so accurate and cautious a pathologist, as M. *Andral* is, should regard an œdematous swelling of the thigh, supervening upon an injury of the foot or leg, as analogous with genuine phlegmasia dolens.

In studying this, as well as every other puerperal, affection, we should never lose sight of the peculiar state of the constitution in which the morbid action is apt to occur; viz. within from one to three weeks after delivery.

The loss of blood, the exhaustion of nervous energy, the excitable state of mind, the secretion of the milk and the systemic disturbance that so often accompanies it, the distention of the cellular tissue, &c. are so many circumstances which must render the puerperal state different from every other, even among females themselves, and which stamp with a peculiar character the diseases that are then apt to occur. No experienced physician will ever lose sight of this most important consideration; and if it had been less neglected than it has been too often during the present century, we should have less cause to lament the not very creditable controversies on puerperal fever, and the melancholy loss of life that has (on some occasions at least) been the consequence.

We have little doubt, in our own minds, that in a large majority, if not in all cases, of genuine phlegmasia dolens, there is some affection of the uterus, cotemporaneous with the outward and visible one of the thigh. We generally find that there is a greater or less degree of tenderness in the corresponding iliac region or even the pubes at the commencement of the attack, and the lochial secretion is always more or less disturbed. That this uterine affection is of an inflammatory nature is more than probable; but then, be it remembered, the character of the inflammation has something unusual and special; it has, if we may so speak, a peculiar idiosyncrasy; and it is this very peculiar idiosyncrasy that makes all the difference—and a most important one it is—between such an œdema of the limb as may supervene upon an injury of the foot, and the proper phlegmasia dolens of puerperal women.

Now, although we confidently deny that phlegmasia alba is either a phlebitis, or an angeoleucitis, or a neuritis, or all three together, we are ready to admit that each or all of these affections may exist simultaneously with it. Such complicated cases are, as a matter of course, more unfavourable than the others, and they will necessarily require certain modifications in the line of treatment to be pursued.

It is certainly much easier to say what the disease is not, than what it really is; and we must frankly admit that its pathology is (in spite of the researches of numerous observers) still very far from being satisfactorily made out.

With respect to treatment, the only safe plan is minutely to examine all the phenomena of each case by itself, and, independently of any preconceived hypothesis as to its nature, to watch its progress from day to day, and to adapt our remedies to the symptoms as they develope themselves, always paying the greatest attention to the state of the uterus, and keeping in mind the peculiarities of the female system in the puerperal state.—(*Rev.*)

---

### Remarks on the Malignant Pustule.

In our last Number we drew our readers' attention to this disease, by several extracts from a Paper on the subject recently published by Dr. *Muller*, and we now avail ourselves of an elaborate memoir by Dr. *Bourgeois*—whose experience must be great, as he has seen upwards of a hundred cases—to give some further illustrations of its history. Dr. B. very properly remarks that the term *pustule* applied to this disease is quite at variance with the phenomena actually observed. It is not a pustule, but a distinct and well-formed vesicle, that forms over the spot where the virus has been inserted; and the contents of this vesicle never assume a genuine or perfect purulent character. The disease is essentially *local* at first; and it is only when the virus has been absorbed into the system, or when the local excitement becomes severe and extensive, that unpleasant constitutional symptoms make their appearance. The result of Dr. *Bourgeois's* experience (and of that indeed of most other medical men) is, that the disease very generally proves fatal if left to its own course; and that recovery is rare, unless when art timely interferes, either to arrest the mischief while it is yet local, or to support and aid the constitution, when it has become *poisoned* with the septic virus. The post-mortem appearances found on dissection do not throw much light on the nature of the disease. This remark, be it remembered, usually holds true of all diseases arising from the operation of a poison, received into the system. The blood has been observed in several cases to be in an unusually fluid condition; and the tendency to speedy putrefaction is often remarkably great—just as it is in many putrid fevers.

Dr. B. gives a very graphic description of the appearances usually presented during life by the face, when the disease makes its appearance there—and unfortunately this is its seat of predilection above every other part of the body. The swelling is often so great that scarcely a single feature can be recognised; the whole head and face looking like a huge distended bladder, with a few depressions or cross lines indicating the situation of the mouth and eyes. Under such circumstances, even when the patient recovers, the deformity left is not unfrequently most distressing. If the lower eyelid be attacked, it often becomes everted; and then we have a red fleshy-looking ring formed round the eye, in consequence of the permanent exposure of the conjunctiva and inside of the tarsus. The upper lid never suffers so much as the lower one. With respect to the nose, the deformity left behind is sometimes still more distressing: for occasionally the whole, or the greater part of, its lobe is utterly destroyed, so that the openings of the nostrils are then like two wide holes in the middle of the face. The lips also may be disfigured at the same time by the formation of scars and the destruction of more or less of their substance. As to the other parts of the face, the cicatrices, though generally less hideous, often give rise to great distortion of the countenance. One source of danger, when the disease is seated in the face, is that the pharynx may become affected; for then the œdematous swelling of the velum and pillars of the throat is sometimes so great as to cause asphyxia.

With respect to the nature and causes of malignant pustule, Dr. *Bourgeois* remarks; " the disease is essentially of a gangrenous and septic character, and is the result of direct absorption of an animal poison into the system. This poison seems to be generated in the bodies of most of our domestic animals—at least in those which are herbivorous, as in the sheep, ox, horse and ass—and is capable of being transmitted by direct contact to mankind. The primary and exciting causes of this epizootic disease among cattle have never been very accurately ascertained. Some writers have attributed it to the nature of the pasturage, and others to an unknown malarious state of the atmosphere."

"Whatever view," adds our author, "we take of the question, experience clearly proves that the disease makes its appearance, in our district at least, almost exclusively during the prevalence of great heats, when the herbage is very dry, and when the cattle are not sufficiently sheltered from the sun's rays. Under such circumstances, we often lose immense numbers of our cattle in this part of the country."

It would seem that an animal may communicate the disease to a human being, although it may be scarcely, if at all, visibly affected itself. It is also by no means uncommon for the poison to be conveyed by insects from the diseased brutes to men. I have had many cases, says our author, of malignant pustule among people who were residing in the neighbourhood of shambles and skin-stores, although they had nothing immediate to do with them.

It deserves to be noticed that some parts of an animal, as the hair or wool for example, retain the poisonous matter for a great length of time, and even after they may have been exposed to washing and other cleansing processes. Dr. *B.* alludes to a case which he once saw in a man, who seemed to have caught the disease by handling the horse-hair taken out of an old sofa.

It has been supposed by some that the mere eating of the flesh of infected animals may communicate the disease; but this is not in accordance with the observations of Dr. *Bourgeois*.

No satisfactory explanation has yet been given why the disease should be so common in some districts, and so rare in others where the climate and soil are much alike. It has been asked, is the disease communicable from one human being to another? or from a human being to one of the lower animals? Dr. *B.* admits the probability of the first case; but knows of nothing as yet to warrant his assent to the second of these queries. We come now to the most important part of our subject, the *Treatment*.

There is no disease in which it is more necessary to have recourse promptly and without delay to the use of proper remedies. We need not allude to the importance, as a preservative and counteracting means, of extreme cleanliness and frequent washing (occasionally with the solution of the chloruret of lime) on the part of those persons, who have any thing to do with infected cattle. Many men expose themselves to much unnecessary risk by putting the knife, with which they have been flaying the carcasses, between their teeth, and by carrying the hides on their bare shoulders. As the disease is strictly and essentially local at first, it is obvious that, if the morbific germ in a part be destroyed before the system becomes infected, the danger is over. The primary object therefore is to destroy the inoculated poison; and this is most effectually done by the judicious application of certain caustics. Dr. *B.* very properly gives the preference to the potassa fusa. The absence indeed of any pustule in those cases, where the eyelids are the parts primarily affected, renders the local treatment under such circumstances very puzzling. At first, all that we can well do is perhaps to apply some stimulating lotion; as for example a strong decoction of cinchona, to which some camphorated spirit has been added; or the swollen parts may be pencilled with the nitrate of silver. If a pustule makes its appearance, the application of caustic is then necessary; but, as a matter of course, it must be used with more than ordinary caution.

Unfortunately in most cases the system is already affected, before we have the opportunity of seeing the case. As to general remedies, some medical men have recommended the use of bleeding and other antiphlogistic means; but Dr. *B.* very properly condemns such practice. The disease is essentially a *septic* contamination of the fluids of the body, and the mere circumstance of there being a hurried circulation and other febrile symptoms is certainly not a sufficient reason to justify such treatment. Even local depletion should be always avoided; for the system will require all the strength that it may happen to have, to enable it to pass through the morbid process; and moreover the bites of leeches are apt sometimes to assume a gangrenous character. Refrigerant and subsequently stimulating and cordial medicines are necessary in most cases; camphor and musk are among the best of this class. The internal administration of the chlorides has sometimes seemed to be of very decided benefit. As a matter of course, much will depend on the strength and healthfulness of the system; and our prognosis therefore must always be very guarded, when the patient has been an irregularly-living or a dissipated character.—*Archives Generales.*

---

### MM. Recamier and Tessier on Gout.

It is M. *Lisfranc,* if we remember aright, who says that the physicians of the Hôtel Dieu in Paris have been long distinguished for the soundness of their practice and the excellence of their therapeutic instructions to the pupils. None deserves this praise better than M. *Recamier,* who has for very many years been recognised by all his brethren as one of the ablest and most successful practitioners in the French Metropolis.

The subject of gout does not often come within the sphere of a hospital physician's prelections; the disease being very generally the penalty of luxurious living. The rarity of the occurrence made us the more anxious to avail ourselves of the present opportunity of making a few extracts (although they are certainly very disconnected) from a clinical lecture recently delivered at the Hôtel Dieu upon the subject.

"The phenomena of irregular gout are often not a little obscure, and it requires all the tact of experienced sagacity to trace them to their proper source. Here is an example.

"A medical man was affected with Amblyopia (imperfect amaurosis) and an insensibility of the right side of the chest. He had also a fixed pain in the nape of the neck; the suffering was much increased on any movement of the part. Besides these symptoms, he experienced occasionally slight flying pains, accompanied with a sense of formication and debility in the limbs; and these were every now and then more or less swollen. The patient's own idea was that there was an incipient caries of the cervical vertebræ; but the real fact was that all his symptoms were the mere outward phenomena of a gouty diathesis. On questioning him, it was found that he had been long subject to pains in the feet; and, upon examining them, it was observed there were traces of tophaceous concretions about some of the joints. A mustard foot bath, and a few glasses of Vichy water, sufficed to bring the pains back to their primary seat; and the patient was thereby soon relieved. We should mention that the suppression of the pain, wherever that was seated, was always followed by a most distressing flatulent dyspepsia....... Not a few cases of hemiplegic paralysis, of cerebral congestion, and even of apoplexy, seem to be decidedly connected with a gouty diathesis...... To define what gout is will puzzle the most ingenious. As to calling it a hyposthenic or a hypersthenic disease, or a neurosis, or by any such appellatives, is absurd. At one time it is inflammatory; at another it is atonic; in one case it is regular, in another it is intermittent; and so on. In short, it is so multiform that it is impossible to say what constitutes it, and what does not."

M. *Tessier*, the colleague of M. *Recamier* at the Hôtel Dieu, appends the following remarks.

"In *gout* we observe various forms, various affections, various morbid products, and various localisations. Under one common name how many diseased conditions, differing from each other in their symptoms and in their seat, are grouped together! In the case of *scrofula* too, we find in the same manner a multitude of diversified phenomena—swelling of the nose and lips, ophthalmiæ, tuberculous deposits in various parts, caries, &c.—occurring in different patients; and yet all these affections are said to be scrofulous. Then again with regard to *syphilis*, the same remark holds true: its proteiform character is unfortunately too well known to every medical practitioner. As the disease makes progress, we observe the morbid products constantly changing their aspect and general character.

"In the case of anomalous gouty complaints, the important matter is to form a correct diagnosis; for, if this be done at a sufficiently early period of the illness, we may pretty confidently assure our patient that we can give him relief, if he will but obey orders. In all cases, where internal organs are suffering, the physician should endeavour to draw the enemy outwardly: it is always well to make him shew his face; for then he can be much more easily dislodged. By attending to this hint, we may often succeed in relieving many serious and most distressing complaints of the head and stomach—forms in which gout often disguises itself—by simply provoking nature to bring on an attack of arthritis. Indeed all the remedies which we are in the habit of employing, such as sinapisms, blisters, alkaline drinks, &c. can only be viewed as means directed against such and such symptoms, not against the real morbid cause or producing element of the disease;—for of that we know nothing."—*Gazette des Hôpitaux.*

*Remarks.*—We do not quite agree with M. *Tessier* (whose observations, however, on the whole we cordially approve of), in his opinion that literally we know nothing of the nature and essential character of gout, and that all we can do with respect to treatment is to draw the disease outwardly to the joints, and to relieve symptoms as they arise.

The disease has always appeared to us to be primarily and essentially dependent upon a disturbance of the digestive and assimilative functions; one result of which is to introduce into the system a chyle that is more or less vitiated, either by the commixture of some foreign material with it, or in consequence of an altered condition of its normal ingredients. When was there ever a case of genuine gout without some pre-existent disturbance of the stomach and bowels? we believe never.

Not unfrequently indeed we meet with persons who will not allow that there has ever been anything the matter with their digestion, although, on further examination, the physician quickly discovers that this is far from being regularly good. Under the universal phrase of being "rather bilious," a vast number of cases of genuine dyspepsia are to be included. The food is imperfectly digested; there is then always a greater or less disposition to the generation of acid in the stomach; and this must be somehow eliminated from the system, otherwise certain morbid actions will inevitably be set up—in one individual, a gouty affection; in another, some urinary complaint; and in a third, perhaps a cutaneous disease. Does this acid ever exist in the blood? M. *Tessier* says that the blood indicates no unusual phenomenon, nor any character that can lead us to believe so. Perhaps not; but this circumstance alone is not a sufficient reason for altogether denying the existence of an acid matter in the fluids. Organic chemistry is in its very infancy, and we are still but at the very threshold of our knowledge respecting the changes which the fluids undergo during disease. The whole subject is full of doubts and difficulties. A few years ago we used to be told that the presence of nitrogen was one mark of distinction between vegetables and animals; and now we find that all plants contain more or less of this principle. What was the reason of this?—simply because one chemist followed the dicta of another, without examining the subject for himself. And so it has been with the examination of the blood and other fluids of the body in a state of disease. Have we any decisive experiments to prove that there is no appreciable difference between the blood of a person in perfect health and of one labouring under well-marked gout? We are well aware of the great difficulty of conducting such experiments, and of the numerous sources of fallacy to which they are exposed; but certainly when we see that a large quantity of acid matter is eliminated from the system, either by the urine or by the formation of tophaceous nodosities about the joints during an attack, we naturally suspect that it previously existed (perhaps not in a free or easily recognised state) in the fluid from which every secretion is derived; and when we find, moreover, that by attention to diet, so as to avoid all indigestible and acescent food, and by the regular use of gentle alkaline remedies for a short time, the tendency to return of the disease is in a great measure prevented, our suspicions surely acquire the force of very reasonable presumptions.

Gout is the disease of all others which should be examined in all its bearings on those broad and comprehensive principles of analytic research, which M. *Liebig* has of late years introduced into the study of animal and vegetable chemistry. The peculiarities of the individual's constitution should be studied; the nature and composition of the food taken should be ascertained; the character and the amount of the excrementitious matters rejected from the system should be examined; and the influence, moreover, of diet on these should be carefully watched and noted. There is something faulty, we may rest assured, at some part or another of the elaborative process, by which the nutriment is converted into, and becomes assimilated with, the circulating fluids; some compound perhaps formed which is not formed in a state of perfect health, and which, acting as a noxious foreign matter, requires to be discharged at some emunctory —otherwise a morbid process, or in other words a paroxysm of gout will be induced. For what is an ordinary attack of gout, but only a crisis of an antecedent illness, or, so to speak, an effort of the system to throw off some peccant matter which has become generated within the body? It would be easy to extend these remarks, and to point out how strictly this view of the disease is in accordance with the results of experience as to the most successful treatment of its various forms; but this we cannot do at present.—*Rev.*

---

### M. Tessier on Hæmoptysis.

There is certainly nothing very original in the following remarks, except perhaps the style of language that is here and there employed. Surely our learned neighbours would do well to use a simple form of speech rather than have constant recourse to phrases which serve to mystify, not to explain.

"Hæmorrhage from the lungs may arise from various causes. Sometimes it is the result of a violent exertion or muscular effort, as running, rowing, lifting a heavy weight, &c. At other times, it is induced by the suppression of an accustomed discharge, as that from piles, &c. In a third set of cases it seems to be idiopathic and constitutional; it may then recur so frequently and so profusely as to cause the sudden death of the patient—as we recently saw in a strong athletic man, in whom however no very distinct lesion of the lungs was discoverable on dissection. In a fourth set of cases the loss of blood is connected with a tubercular state of the lungs; this complication is unfortunately the most frequent occurrence of all.

"Under the circumstances first alluded to, the treatment is very simple; the medical man should not be over officious; there is often no occasion for having recourse to bleeding or other active measures; all that is necessary is to enjoin great quietude for a time, and to use such means as are best suited to prevent the recurrence of the hæmorrhage. If, however, there be a tendency to its return, it will very generally be proper to draw blood, unless there be some well-marked contra-indicating circumstances to dissuade the practice. For example, if the patient be at all phthisical, bleeding is to be used only with the greatest reserve, even although the pulse at the time be hard and concentrated. A most important therapeutic principle, with respect to the employment of this remedy, is that it should be in all cases subordinate to, and regulated by, the condition of the patient's constitution.

"Certainly the most precise index, as to the state of the vital forces and the functions of organic life, is the pulse. If this be hard, and if at the same time the disease is still in the state of concentration, there is a strong motive to draw blood, in consequence of the existing plethora, the exaggerated sanguification, and the tendency there must be to congestion in different parts of the body. By relieving the excess of the circulating fluids, we induce a critical change which is often most salutary.

"But, in place of there being any genuine plethora, we may have to do with a case where the hæmoptysis co-exists with a diminution in the energy of the vital forces, or again with a simple oppression of their activity, or lastly with a genuine atony of the whole system. In such circumstances, we should carefully examine the existing state of all the great organs of the body, in order that we may discover how each function is performed; as it is not unfrequent to find that a disturbance of the stomach, or liver, or kidneys for example, has had something to do with the pulmonic affection. If there is a decided saburral state of the stomach present, we should have recourse to an emetic. *Stoll* observed great advantage from the practice in a number of cases, to which he gives the name of bilious or gastric hæmoptysis. By getting rid of the gastric disturbance, we remove one of the principal elements of the disease, and we reduce it to a state of greater simplicity. Having destroyed one of the morbid states, we attack the other by means of bloodletting and other remedies."

M. *Tessier* then describes what he calls a nervous form of hæmoptysis, which is apt to occur in highly excitable females. His remarks however on this subject are exceedingly unsatisfactory and obscure: we suspect that he describes the disease rather from fancy than from actual observation. Does he allude to the not unfrequent form of pulmonic hæmorrhage that is liable to occur, when the catamenial secretion is very irregular or perhaps totally suppressed? If so, we can understand him; but not otherwise; as we never saw a case of what we should think of calling nervous hæmoptysis. The following passage may possibly be meant to apply to the amenorrheic form of the disease.

"The hæmoptysis, which is apt to occur in anæmic subjects, requires the exhibition of steel, bitters, and balsamic medicines. We must be careful not to confound such cases of passive hæmorrhage from the lungs with that form of the disease, which is connected with the presence of tuberculous deposits. In passive hæmoptysis, the blood rejected is usually of a black colour, diffluent and not so plastic as in health; the pulse too is usually soft and weak, and the muscular system flabby."

M. *Tessier* then treats of an *intermittent* form of hæmoptysis, which requires the use of bark for its cure; and closes his general remarks by acknowledging that there is sometimes no little difficulty in forming a correct opinion of the pathology of pulmonary hæmorrhage, and in determining the exact nature of the prevailing morbid element in each form of it. He insists particularly on the necessity of perseveringly continuing any mode of treatment for a length of time; and, by way of illustration, he alludes to a curious case in the practice of his experienced colleague M. *Recamier*, who, by bleeding a patient *three hundred times (!)* in the course of two years, succeeded in finally subduing "une affection des plus rebelles."—*Gazette des Hôpitaux.*

### Remarks on Phthisis.

There is much good sense in the following clinical observations, by the physician of the Hôtel Dieu, on this, alas! too well-known disease of every country.

"It is important to discriminate correctly the different periods or stages of phthisis, and to distinguish the various forms under which the disease is apt to appear; as much depends on the first of these considerations how our prognosis should be formed, and on the second, what remedial means hold out the best promise of relief, if not of cure.

"Each of the periods of phthisis has its peculiar therapeutic indications that require to be fulfilled; and it not unfrequently happens that there is a contradiction between those pointed out by the local, and those by the constitutional symptoms.

"The age too of the patient is a very material point to be taken into account, in our prognostic consideration. As a general remark, it may be said that there is much greater chance of arresting the course of phthisis after the age of 35 years, than at an earlier period of life. We not unfrequently observe that, after the 'mezzo cammin di nostra vita,' the disease remains stationary for a length of time; and its duration may then be protracted for two, three, or even more years. But even under such circumstances as these, a real cure is seldom or never effected; the progress of the disease may be retarded, but it is no less surely fatal at last. We may soothe the suffering; we may check the symptoms as they arise; but all the while the weakness goes on increasing, and the vital energies are becoming more and more exhausted. The patient himself, perhaps, will not admit this; but his attendants too distinctly recognise the melancholy truth, and are forced to allow that, although their friend is much easier than he may have been, he is not at all capable of standing more fatigue—quite the reverse.

"If the medical man does not bear this constantly in mind, he may allow himself to be deluded by the reports made to him and, thus fall into the unpleasant mistake of believing that there is a positive amendment—even up to the very day before dissolution. It is necessary for him not to permit his hopes to at all control his professional judgment; otherwise he will most probably be much blamed afterwards for his want of sagacity.

"There is thus often very little or no correspondence between the condition of the local and that of the constitutional disease: the latter may be much modified by appropriate treatment, while the former is gradually becoming worse and worse. How rare is it that genuine vomicæ or pulmonary caverns ever undergo any salutary change! They may, indeed, under the influence of certain medications, contract somewhat in their dimensions, and the nature of their contents may become altered; but any real and permanent amelioration is not to be expected.

"Nevertheless, it is the duty of the physician never to neglect the use of any means which may serve to allay each symptom of distress as it may arise. Much may be done in this way, to prolong the life and soothe the suffering of the patient."—*Gazette des Hôpitaux.*

*Remarks.*—It has often occurred to us that medical men generally overlook far too much, in the treatment of such prolonged and distressing a disease as pulmonary consumption, the influence of the moral and religious feelings upon the progress and duration of the morbid changes. He, indeed, must be little of an observant practitioner, who has not remarked how much more rapid the course of one case is than that of another—quite independently of the extent of the existing lesion of the lungs, or of the general strength of the patient's constitution.

Now what is *the* cause—or, to speak more guardedly, one of the causes—of this difference? Is it not, (often at least,) the state of the sufferer's mind and feelings? and does not much, very much, depend on the simple circumstance whether these be calm and patient, or restless and unsubmissive?

The man, who regards every bodily affliction, not as a casual or chance occurrence, but as a chastening visitation of a Higher Power to try his faith, or to wean his worldly attachments, and who submits to them with a becoming composure, has a mighty advantage over him that views these things differently. Not only are the bodily sufferings of the former unquestionably less hard to bear at the time, in consequence of the greater tranquillity of his feelings, (for who has not found, in his own experience, that pain is worst borne when the mind is unhappy—

Mensque pati durum sustinet ægra nihil)

but the very morbid change that is going on within the chest, is found to advance much more slowly and gradually to its fatal issue. And surely there is nothing to surprise us in this. Whatever disturbs the evenness of the circulation, will be observed to promote the progress of any structural disease: and how can it be otherwise? Do we not constantly find that, if there be any febrile re-action in the system, such processes as those of suppuration and ulceration are greatly accelerated? and is it not the daily practice of the experienced surgeon to use every *physical* means in his power to moderate and subue such reaction after operations and accidents?

Well, the restlessness of the feelings is a fever of the mind; and this cannot long continue without inducing its namesake condition in the bodily frame. The subject of the mutual re-action of mind and body is one of the most important that can engage the attention of medical men; and yet it has never been treated as its value demands. We are all too much taken up with the *physique*, and too little with the *morale* of our therapeutic resources; and hence it is that most of us seem to think that we have amply fulfilled all our duty, if we but use our best skill in selecting from the materia medica the most appropriate remedies.

As a matter of course, we cannot enlarge upon this subject at present; but we are unwilling to lose the opportunity of expressing our own opinion of the vast importance of a religiously tranquil state of the mind in moderating the progress of organic disease, and therefore in prolonging life, as well as in blunting, at the time, the sharpness of all those aches and ills which flesh is heir to.—(*Rev.*)

### Cases of Pleuritic Effusion, with Remarks.

In the present dearth of novel and instructive matter in the Continental Journals, we select the reports of two cases of this very serious affection, which we find recorded in the clinical lectures of M. *Tessier* at the Hôtel Dieu. On no disease has the improved pathology of modern times thrown more light than on this. Its diagnosis may now, in most instances, be satisfactorily established by means of auscultation, and we are moreover possessed of several potent curative means, if there has been no unnecessary delay in having recourse to them.

*Case* 1.—The first case occurred in a woman, who was admitted into the hospital with pleuro-pneumonia on the left, and simple pneumonia on the right side. The inflammation of the substance of the lungs was quickly arrested by bleeding, &c. but the pleuritic symptoms did not so readily yield, and effusion into the left thoracic cavity was the consequence, so copious that the heart was pushed over to the right side. Repeated blistering and the use of purgative and diuretic medicines failed to cause the absorption of the fluid.

The case had been in this state for about a month, when it was determined to make a trial of galvano-puncture. The two first applications had a remarkable effect; for there was almost immediately afterwards found to be a diminution of the effusion to about four finger-breadths below the clavicle, the resonance on percussion having again become normal over this extent. After several other applications of the Galvano-puncture, the resolution of the disease appeared to be almost complete; the resonance being re-established over more than two-thirds of the chest. The patient, feeling himself to be so much better, insisted on leaving the hospital before a perfect cure was effected.

*Case* 2.—The pleuritic effusion in this instance, also, was on the left side, and had supervened in a very similar manner to what took place in the preceding one. There was a visible enlargement of the left side; no respiratory murmur could be heard over it, and the sound on percussion was uniformly very dull, even in the sub-clavicular region. Symptoms, however, of a partial resorption began to make their appearance, and hopes were therefore entertained that the lung was not so much shrunk and contracted in volume, as it is usually found to be when the entire cavity of the chest has been filled with fluid—a state of the lungs that requires a long time before they recover their natural dimensions, even after the effusion has been dissipated.

It is in consequence of such an occurrence as this—the non-expansion of the lungs after the resorption of a copious pleural effusion—that many of those depressions and irregularities of one side of the chest, which are not unfrequently observed in deformed children, are to be traced. We have very little doubt, says M. *Tessier*, that, if the development and progress of many cases of deformity in children were attentively watched, it would be found that they were often attributable to a pleural effusion that had become rapidly absorbed, while the lung had never recovered its normal dimensions.

This state of things is also not unfrequently observed in women—who, it should always be remembered are, in many respects, like children, both in their organisation and in the diseases to which they are liable.

It is often stated, in books and lectures, that pleurisy is not a grave disease, and that it will generally give way to very simple treatment. This is a great error; inflammation of the pleura should never be regarded otherwise than as apt to be very serious in its consequences.

The treatment of the disease, when at all severe, will often require much practical discretion. For if bleeding and other depletory measures be carried too far, or their use continued too long, the tendency to effusion will be increased, while the active powers of the system may be too much lowered for the necessary absorption to take place with sufficient promptitude.

It is sometimes not an easy thing to determine the exact time when the change of treatment should be made. Now, it is in such cases, that the real value of auscultation cannot fail to be appreciated. If the effusion has already become very considerable, and the vital power has been much reduced, before a correct diagnosis has been formed, the medical man will be placed in a very difficult and often most perplexing situation. The time for the further employment of antiphlogistic remedies is past, and yet the use of stimulants is not unattended with danger.

A fatal termination may be the consequence of an officious or mistaken treatment, as well as of the progressive aggravation of the disease, or of an inflammatory attack on the other side of the chest, with or without effusion at the same time. There is always very great danger in such cases as these. The fluid may, indeed, be withdrawn by operation; but the lung will probably not be in a state to recover its normal expansiveness, and the respiration is, therefore, little or not at all relieved. Perhaps, too, air may find its way into the pleural cavity; symptoms of an inflammatory and suppurative character will then supervene—a state of things that is almost inevitably followed by death.

Occasionally, indeed, the purulent matter, that is secreted in the pleural cavity, makes its way into the air-cells and tubes of some part of the lungs, and is rejected by expectoration: immense quantities have been known to be discharged in this way. But, unless the patient's constitution is unusually robust and elastic, there is but a very slender hope of his recovery under such circumstances. In other cases, of however still more rare occurrence, an abscess forms in the side, and the pleural effusion finds an escape outwardly.

It is, therefore, obvious that the prudent physician will never be hasty in giving his prognosis in cases of chronic pleurisy, even although the existing symptoms may not indicate any unfavourable issue.

We may here add that this experienced physician, in his clinical lecture, makes a remark, the truth of which will be at once recognised by all men of experience.

"Whenever in a chronic affection, such as we have been treating of, you find every now and then occurring an exacerbation, or a fresh attack, of pyrexial symptoms, you may at once regard it as a most unfavourable omen, and suspect that there is some deep-seated lesion or another of the organization. As this was the case in the present instance, I without delay consulted M. *Recamier* as to the propriety of withdrawing the thoracic fluid, by performing paracentesis of the chest. Unfortunately for the credit of this operation, we are too much in the habit of delaying its performance, till the patient is in imminent danger. On withdrawing the trocar, from four to five *litres* of a puriform fluid escaped. For some days afterwards the side was quite resonant on percussion, although the lung was certainly not expanded during breathing, for no respiratory murmur could be heard. It is therefore probable that there was a certain amount of air (whether this had entered by the canula, it is not easy to say) between the two pleuræ. The symptoms went on, upon the whole, favourably. The lung seemed to be beginning to recover its expansibility; a slight murmur was heard in the sub-scapular region; and the ribs moved upwards and downwards, during the acts of respiration, more than they did before. As a metallic tinkling was occasionally perceptible, and succussion of the chest produced the sensation of a wave of fluid, there is every reason to believe that a considerable quantity of effusion is still present."—*Gazette des Hôpitaux.*

---

### CASE OF HYDRO-PERICARDIUM: PUNCTURE AND EVACUATION OF THE FLUID, &c.

A youth, 19 years of age, was seized with sharp pain in the right side of the chest, great dyspnœa, and other signs of pneumonia. He had been ill for some time when he was taken to the Vienna hospital; the active stage of the disease had already passed over, and was succeeded by the signs of effusion within the thorax. The face had become puffy and œdematous, and expressed intense anxiety; the breathing was short, hurried, and accompanied with a rattling noise; there was a frequent cough, which brought on a pungent pain in the left side; the left jugular vein was much swollen, &c. On percussion, the whole extent of the sternal region of the chest was found to be very dull; under the left clavicle however, and along this shoulder on to the axilla, the sound elicited was clear; but again it became dull over the lateral region of the chest. On the right side, the resonance was normal, except at the lateral parts from about the fourth rib. The liver was found to extend beyond the edge of the false ribs, for at least two finger-breadths. The impulse of the heart was very feeble, and the sounds of its action very indistinct. There was a strong respiratory murmur (the expiratory one being unusually loud) heard over the whole extent of the left side, except in the cardiac region, and also over the front of the right side: lower down, however, it was scarcely audible. The pulse was rapid, small, and irregular; the urinary secretion was scanty and deep-coloured. The patient complained of a continual sense of pressure at the epigastrium, especially when he lay on the left side: firm pressure over the heart gave rise to a sharp pain in that region.

The diagnosis formed was that there was a considerable effusion within the pericardium—the consequence of pericarditis—causing the compression of the inferior lobe of the lung; also slight exudation in the right pleura, with an infiltration of the pulmonary parenchyma, the result of pneumonia; general bronchial catarrh.

As symptoms of a peritoneal effusion made their appearance in the course of a few days, it was determined to have recourse to *paracentesis pericardii* without delay.

The puncture was made (5th August) in the fifth intercostal space, about two inches from the left extremity of the sternum, and about one inch below the mamma—in order to avoid more certainly wounding the internal mammary, or any of the great vessels. At first, only a small quantity of reddish fluid escaped by the canula; but, by using gentle compression, as much as three pounds (livres) was discharged. The patient experienced great relief; and the double sound of the heart, as well as the "bruit de frottement," were heard much more distinctly immediately afterwards. Next day, symptoms of inflammation in the lower lobe of the left lung made their appearance: the patient was therefore bled. For several successive days, there was a manifest increase of the pericardiac effusion. Digitalis and iodine were administered, and mercurial frictions were employed at the same time.

As the disease was manifestly making progress, a second operation was performed, (22d August); but on this occasion not more than three-fourths of a pound of fluid flowed out. Again there was an attack of pneumonia; but it was fortunately subdued without much difficulty. The state of the patient seemed to improve until the 4th of the following month, when the dropsy of the pericardium and also of the peritoneum began rapidly to increase. Death took place on the 12th.

*Dissection.*—Adhesions of the right pleura, and considerable effusion in both cavities of the chest—to the amount of between eight and nine pounds (livres) on the left, and of about five on the right, side: both lungs compressed and flattened against the spinal column, and several patches of tuberculous deposit throughout their substance. Pericardium adherent to the heart over a great extent of its anterior surface; and several ounces of fluid within its cavity. The heart itself large and flabby; its cavities, especially those on the right side, considerably dilated, and filled with coagulated blood. From 15 to 16 *litres* of serosity within the peritoneum; this membrane somewhat thickened in several parts; the liver enlarged, dense, and of a brown-red colour.—*Schmidt's Jahrbucher.*

*Remarks.*—Most British physicians will unquestionably disapprove of the practice adopted in the foregoing case. No advantage could be reasonably expected from discharging the pericardiac fluid, while both cavities of the chest contained so much. We are not aware that paracentesis of the pericardium has ever been performed in this country. Without going so far as to reprobate the operation under any circumstances, the cases, we should think, in which it is justifiable, must be of exceedingly rare occurrence. It is a great error with many medical men to be desirous of doing something in every case: they seem to forget that there is quite as much skill required in knowing when to do nothing, as to employ vigorous treatment when this is called for.

*A propos,* we remember an anecdote of a clever but sarcastic surgeon, which may deserve reporting. On one of his colleagues, who was rather a busy meddling sort of practitioner, remarking, in reference to a dangerous case in the hospital, that in his opinion such and such were the "indications of treatment" to be pursued, he very coolly observed that, in his opinion, "the only indication was death." The lesson suggested is a useful one: *ne quid nimis.—Rev.*

---

### ANIMAL MAGNETISM, A FASHIONABLE INTERLUDE IN PARIS.

Our lively neighbours cannot live without their jest, whether this be in the form of a mere *jeu-d'esprit,* or of a *spectacle* of some sort or another. If there has been a great fire in Paris (destroying perhaps a vast deal of property, not to mention life), it is more than likely that the ladies dresses in the ensuing season will be of flame-coloured silk; and if cholera, or any other novel scourge, makes its appearance among them, we are sure to hear of the fashionable hats, bonnets, shawls, &c. being *à la cholera, à la grippe,* &c.

One of their favourite themes of sport in the present day seems to be Animal Magnetism. The following extract, from a very sensible article in a recent number of the French Medical Gazette, may probably amuse our readers.

"If this kind of charlatanism (the puffing of ignorant empirics) is disgusting in consequence of the serious injury to health that is often the result, there is another kind which, although less immediately hurtful to society, is now spreading upon a broader scale and is likely to be ultimately attended with still more serious mischief; we allude to *Animal Magnetism.* This doctrine, utterly rejected by the Academy, and abandoned, at least ostensibly, by the intelligent *Savans* who embraced it a few years ago, has now fallen into the hands of a set of the lowest empirics, who seem determined to turn it to the most profitable account. Several of these *industriel* rogues are in the habit of exhibiting in the saloons of Paris pretended somnambulists (of both sexes), who perform before the astonished spectators various mountebank tricks for the small sum of from 20 to 30 francs each performance! There is now scarcely a *soirée un peu sortable,* which has not its little somnambulic representation. And yet, in the name of all that is wonderful, these poor devils, whose address certainly is not a whit beyond the tricks of the most common juggler at a fair, are received into the most splendid drawing-rooms of the metropolis, amidst the most polished society of Europe—(well done, Monsieur Français! your nationality is always amusing)—as marvellous and exceptional beings, whose words are received as the responses of an oracle, and whose acts are almost believed to be supernatural!

"We have been present at some of these mystifications, and we fairly confess that we knew not whether to wonder most at the unheard-of impudence of the performers, or the extraordinary credulity of the spectators. And strange to believe! this is going on in the nineteenth century, in the midst of all that is most enlightened in literature and science, without opposition or reclamation.

"Besides these public exhibitions, we have private ones, got up at so much an hour; and then there are occasionally medical consultations—to the disgrace of our profession too often sanctioned by medical men. In short, there is not a street in Paris that has not its somnambulist juggleries.

"Religious fanaticism has been for some time past availing itself of animal magnetism, as an apt means to impose on the credulity of the ignorant; for it is not difficult to perceive in the pretended miracles—which after so many years of interruption, have lately began to make their re-appearance in different countries in Europe—the influence of a superstitious belief once more brought into fashion by the modern disciples of Mesmer. The Church, it is true, has condemned the practice of Animal Magnetism, but it should be remembered that it has never denied the truth of the somnambulic exhibitions. Hence it comes that, while many attribute the phenomena to the operation of some immaterial fluid or to some particular condition of the nervous system, others are convinced that they are the work of the Devil himself. It is this latter opinion that seems to be the one that is generally received. We cannot wonder at this, when we call to mind that the bulk of mankind have always had a mighty partiality for whatever savours of the preternatural and miraculous. Hence it comes that now-a-days not a few cases of catalepsy, extasis, hysteria, mental hallucination, &c. are put down to the agency of demoniac *possession*."—*Gazette Medicale.*

*Remarks.*—It is rather curious to observe what has been going on in our own country about this mountebankery of science, and to notice the different views taken of it by the Clergy. While Mr. *M'Neile,* the well-known eloquent minister of Liverpool, has published a sermon "On the Satanic (no mincing word, certainly) Agency of Mesmerism," a pamphlet has recently appeared from the pen of a clerical brother, entitled "Mesmerism the Gift of God!"

Who shall decide, when doctors disagree?

---

### Remarks on Italian Medicine.

An intelligent French physician, M. *Combes,* has recently paid a visit to Italy, with the view of examining into the state of medical doctrine and practice in some of the leading schools of that interesting country. We have not yet seen the original work, but, meeting with a copious review of it in one of the Paris journals, we deemed it worth while to make the following extracts from it.

"*Hippocrates* has truly observed that 'in general whatever the soil of a country produces is conformable to the country itself.' This remark is quite as applicable to the inhabitants, as it is to the vegetable productions, of a country. The character and temperament of man are in no slight degree influenced by the medium in which he is born and developed; his bodily health, as well as the passions of his soul and the faculties of his mind, are all more or less under the control of the physical circumstances in which he is placed. If he is the king of nature, his sovereignty resembles that, not of an arbitrary or despotic, but of a constitutional, throne, hemmed in by charters and by parliaments; and the power that he wields is ever under the weight of legal restrictions. How little suffices to alter the character and the energies of human beings! A few additional degrees of temperature, the exposure or non-exposure to a certain wind, the nature of the soil on which they live—these, not to mention many other circumstances, are found to modify not only the character and manners of a people, but even the vigour and the power of their intellect. You aspire to the glories of science, and the atmosphere which you breathe condemns you to become an artist; your mind dreams on discoveries of practical philosophy, and pants to tread the path of calm observation, and you find that, in spite of all your wishes, your imagination cannot resist the allurements of nature around you, but plunges into the fairy domains of ideality and romance."

"Under whatever clime human beings come into this world, they all bring with them at birth the same constitution of moral development; the same faculties lie dormant in the souls of all. These indeed are unequal from the first in different persons, in consequence of certain hereditary elements which stamp a peculiarity on each nation, each family, and each individual; and this inequality will be more and more strongly marked according to the influences of climate and of early education. This is the reason why nations, although possessing most of the attributes of intellectual greatness, are nevertheless distinguished from each other by the special development of one faculty in a predominant degree. In all ages have the Greeks been pre-eminent for the fervour of their imagination, and the inhabitants of Britain for their cold and prudential reason.

"Nations, like individuals, have therefore their characteristic temperaments or idiosyncrasies of constitution. Now the temperament of Italy is its ideality; its very existence is eminently artistic. Living in a region clothed with light (*vestitos lumine colles)*, bathed in a warm and balsamic air, and breathing an atmosphere of romantic associations, little is the wonder that its inhabitants are so distinguished from almost every other people for the richness and grace of their sensual conceptions. Every thing in nature around them leads the mind to indulge in images of softness and beauty, and to withdraw it from abstruse and elaborate studies. Now what is the result of this tendency in reference to scientific pursuits? In schools, where pupils and masters alike have all more of the poetic temperament than they are themselves aware of, the character of the instruction will certainly be more or less dogmatic; and why?—because implicit faith is a positive *besoin* of such a mental organisation. Such a state of mind naturally leads on to a taste for generalizing on all subjects, and this very tendency is promoted by the copious richness of their sonorous language. Thus it is that the systematic conceptions of the Italians, proceeding from deep conviction, lead to exaggerated applications in theorising—and often too to an inordinate rashness in practice.

"The characteristic features of French medicine in the present day are a sort of disrelish for all systems, and a taste for the elaborate investigation of every problem of enquiry. Hence the practice of rigorous and oft-repeated observation, the numerous experiments, and the constant recourse to the aids of the scalpel, the test-tube and the microscope. Indicative of the same thing is the subdivision of science into a number of different branches. Each of these is pursued with indefatigable zeal by its different votaries.

It is scarcely necessary to allude to the characteristics of German medicine: the same laborious curiosity and indefatigable research, the same fondness for mystical speculation, the love of the marvellous and extravagant in physics as well as in metaphysics—these long have been, and still are, the prominent features of the German school." * * * * *

In Italy at present (according to the observations of M. *Combes*) there are very few men, who apply themselves with diligence to the patient investigation of details upon any particular subject of enquiry. Most of the physicians have adopted certain general principles of medical doctrine; and on these they base their practice. Hence it is that almost all their treatises assume the synthetic form, and that, even in their works of elementary instruction, the exposition and description of details are too often sacrificed to the abstract generalities of an ingenious dogmatism.

But let it not be forgotten that we are talking of a country which has produced a *Spallanzani*, a *Morgagni*, a *Scarpa*, &c. and can still boast of *Bellingeri*, not to mention other distinguished names of medical literature. Yet, in spite of these eminent exceptions, it cannot be denied that many of the branches of professional knowledge have made but little progress, for very many years, on the other side of the Alps.

Physiology has retained pretty generally a purely dynamic character; the classical work of *Medici* does not even mention a vast number of well-established facts, and the same remark holds true of the lectures of Professor *Martini* at Turin. If such be the state of Physiology, or that branch of medical science that treats of the functions of the body in a state of health, we cannot be surprised that those of Pathology and of clinical medicine are very faulty and defective in many respects.

"In some districts of Italy, and especially at Naples and Modena, Hippocratism is altogether prevalent. In the latter city, indeed, the Faculty of Medicine may be considered as an association less for instruction in modern medicine than for perpetuating the doctrines of the Coan sage. The orthodoxy of the school is based upon them; everything is redolent of antiquity; the students are taught to regard the Aphorisms as the oracles of medical truth; when they aspire to the doctorate, they are called upon to expound and illustrate one or more of these pithy sayings; and it is with their hands, laid upon the works of the old Greek, that they take the oath upon the day of their inauguration.

"While the doctrines of the Hippocratic Medicine, more or less liberally interpreted, are almost universally received through the whole of the South of Italy, the northern half of the Peninsula has been long agitated by the disputed tenets of Contra-stimulism. Turin, Pavia, and especially Milan, are the head-quarters of this school. *Rasori* its founder started, it is well known, as a disciple of the Brunonian doctrines, and was the translator of the Scotch physician's work. All the phenomena of disease he strove to reduce to one simple morbid action, viz. excessive or deficient stimulation; and, like every other advocate of an exclusive system of pathology, he fell into the most egregious blunders, which proved to be injurious alike to sound theory and to safe practice. All diseases he considered to be systemic or general; and local inflammations therefore be looked upon as mere complications or accidental phenomena—the very reverse of the Broussaian creed. Professor *Tommasini* of Parma adopted a sort of intermediate doctrine between these conflicting systems. Assenting to the general principle of his fellow-countryman *Rasori*, he was too sagacious not to observe that local irritation, when extensive or long continued, is apt to give rise to constitutional diseases. Let us not deprive this distinguished physician of his due; it is right to acknowledge that he clearly enunciated the importance of local affections, as the cause of many diseases, before *Broussais* had published a line upon the subject."

"One of the most eminent physicians of Italy in the present day is M. *Buffalini* of Florence. His clinical lectures draw a large concourse of students, and his fame nearly equals that of his distinguished rival of Parma, *Tommasini*. He is generally regarded as 'un medecin organicien;' and yet his lectures and practice savour strongly of the Essentialist school. In commenting upon any case of fever, he pays minute attention to every appreciable alteration alike of the fluids and of the solids, and he does not hesitate to acknowledge openly that the changes observable, either in one or in the other, are by no means uniformly in accordance with the severity of the constitutional affection, or can be considered as the proper *causes* of it. In many respects he is a decided Humoralist; but, while leaning strongly to the opinion that most fevers are attributable to certain changes in the circulating fluid, he on the whole does not trouble himself much with prying into the nature of morbid individualities, but contents himself with scrutinising their exciting causes, the various symptoms which they exhibit, and with devising the means best fitted for their relief. Every one, who has had an opportunity of witnessing his practice, speaks in high terms of it: avoiding the pernicious errors of Contra-stimulism, (which he has contributed in no small degree to throw into discredit,) he shews great discretion and tact in the use of his remedies. He has published an able work entitled '*Des fondemens de la Pathologie Analytique.*'

"Italy is far behind France in many of the departments of medical science, and certainly in none more so than in Chemistry. There are no good laboratories for the preparation of medicines, in almost any part of the country; and hence little or no progress has been made in Toxicology, although the writings of M. *Orfila* have been long highly appreciated. What will probably surprise most travellers, as much as anything, is to find that no correct analysis of the numerous mineral waters of Italy has been made by any native physician.

"Italy possesses more journals of medicine and pharmacy than France does; (we certainly were not prepared for this announcement;) but the division of the country into so many separate states places a great bar to their diffusion and general usefulness. The medical press suffers much from the consequences of the general political organism of the country; it has not a few obstacles to overcome, and a good many dangers to avoid. Thus, for example, when Homœopathy first made its appearance at Milan, under the patronage of the Austrian government, the subject, it was soon found, could not be discussed in all its bearings with perfect freedom. The whole business of journalism is fettered and cramped by restrictions and vexatious annoyances. Hence it is that scarcely any of the periodicals bears that stamp of originality and freedom of opinion, which constitute the main utility of such publications; and that their chief contents are mere compilations from the French, English, and German journals. As long as such a state of things lasts, we cannot look for that energy and devotedness in the cause of professional advancement which we find in the writers of France, Germany, and Great Britain. Surely medical science may fairly be regarded as a free and open field for unlimited boldness of speech and enquiry; but, alas! it has always been found that tyranny is never satisfied until it has brought everything under its iron yoke."—*Gazette Medicale.*

---

### Statistics of Insanity in France; Influence of Civilisation.

The following letter was read by M. *Briere de Boismont* at a recent sitting of the Royal Academy.

"Five years ago, I had the honour of reading a paper before this learned assembly upon the influence of Civilisation on the production of Insanity, and I then endeavoured to shew, by reference to numerous tables, that the frequency of this most melancholy affliction increases proportionately with the advances which a nation makes in social and mental attainments. Whatever opinion may be formed on this subject, no one can dispute the fact that moral causes give rise to alienation of mind more frequently than those of a purely physical or bodily nature. In 1807, *Pinel* distinctly enounced the truth of this position. Out of 683 cases, which had occurred within his own obervation, as many as 464 were clearly attributable to moral causes. *Esquirol* too has added his valuable testimony to the same effect; for out of 274 cases, which he has reported, 167 were traceable to the influence of psychical disturbance. According to the recent very elaborate researches of M. *Parchappe,* the relative number of cases arising from mental and from bodily causes is in the ratio of 63 to 37—the first being therefore nearly double the second.

"If then the influence of the one cause be so much greater and so much more generally felt than that of the other, we cannot be surprised to find that the frequency of insanity should increase with the advances of civilisation, more especially in those countries where the mind is more worked and excited than elsewhere.

"It is exceedingly difficult to obtain exact reports of the number of the insane in different districts of France. Although Government has expressly ordered that each department should have its separate institution, this is still but very imperfectly carried out in various parts of the country.

"M. *Guislain,* whose excellent works on mental disorders are universally appreciated, has stated, in his official report published by order of the Belgian Government, that the number of the insane in that country (as far as can be ascertained) is 5105, out of a population of 4,165,953 inhabitants; but he adds that this number gives only about two-thirds, or so, of the entire amount, in consequence of the number of insane persons dwelling in boarding houses, convents, &c."

M. *Boismont* calculates the number of the insane in France at nearly 30,000.

---

### The Geography of Diseases.

During the course of the present year, there has been published in Paris a work, entitled An Essay on Medical Geography, or Studies of the laws which preside over the Geographical distribution of Diseases, and of the Topographic relations existing between them; the Laws of Coincidence and Antagonism, &c.

The subject is certainly a good one for a clever man to write upon, as the field is scarcely at all pre-occupied, and the information communicated may therefore be expected to be more novel and attractive than what forms the staple of most modern medical works. We are indebted to the pages of the Gazette Medicale for the following notice of M. *Boudin's* Essay.

"The history of diseases has its Chronology as well as its Geography. On the first of these points, M. *Pariset* has recently made some very ingenious observations, in the way of preface to his Exposition of the Modern Origin of the Plague. Some of the German physicians have enlarged much on the doctrine of morbid metamorphoses or transformations, and have endeavoured to apply its principles to the elucidation of the development and diffusion of other diseases.

"The domain of Medical Geography is already rich and extensive, and travellers are yearly adding to its resources. M. *Boudin*, the author of the work before us, first announced the germ of his ideas in his Treatise on Marsh Fevers, and it is most gratifying to know that there seems to be every probability of these being now fully carried into effect, since the recent institution of "*medecins-voyageurs*" by the Royal Academy.

Botanical writers may justly claim the merit of having first directed the attention of philosophers to the importance of geographical position as an important element in scientific enquiries, by pointing out the influence of climate on the distribution of plants, and thereby proving the sympathy, so to speak, of some, and the repulsion of other, general species for each other.

M. *Boudin* frankly acknowledges the source whence he derived the first hint of his ideas; and he adopts nearly the same division of geographical causes or agencies in reference to diseases, which several botanists have established in reference to the vegetable kingdom. These are—1, the Latitude and Longitude; 2, the Elevation above the level of the sea: and, 3, the Geological character of the soil.

The first of these causes—the latitude and longitude of a place—exercises a very marked influence, not on the character or form only, but even on the very development and existence, of certain morbid phenomena.

The Yellow Fever, the Plague, and the Cholera, have their peculiar and well-defined theatres of action, at least in their state of *endemicity*: these theatres being the deltas of three great rivers, (the Misissippi, the Nile, and the Ganges,) in the hot regions of the earth. With certain modifications, the peculiar nature of which has not hitherto been well explained, the *embouchures* of other rivers, and generally indeed all marshy countries, are observed to be the seat of certain fevers which are characterised by having an intermittent or remittent type. It is for this reason, among others, that M. *Boudin* has been led to conclude that there must be a sort of family resemblance, so to speak, between the Yellow Fever, the

Plague, and the Cholera on the one hand, and Paludian Fevers on the other; and he goes so far as to conjecture that all these diseases are to be regarded only as different manifestations of one and the same morbid agency. Among other circumstances, he points out a very curious feature of resemblance which is said to be common to them all, viz. their power of excluding pulmonary consumption and typhoid fever—diseases which, *en revanche*, exercise their destructive sway over regions that are not marshy or burned up with a tropical sun.

The great *Humboldt* has remarked, in his most valuable work on South America, that the highest limit of the *melastomatous* tribe of plants is likewise found to be that of the Yellow fever: it would therefore seem, that its morbific germ does not reach higher than a certain elevation above the level of the sea.

The plague, too, shews a somewhat similar character; for it has been remarked at Cairo that, while the lower quarters of that city are grievously afflicted with it, the citadel is usually quite exempt from its influence.

At Constantinople, also, it is noticed that it seldom reaches as high as the elevated points of the seven hills, on which this Empress of the East is built. Nearly the same thing may be said of the *Endemic*, but certainly not of the *Epidemic*, Cholera.

These facts, therefore, clearly prove that the elevation to which the morbific germs of certain diseases are usually carried in the atmosphere, is by no means considerable. If space permitted us, we might here show that the germs of other diseases are limited to a very small circuit around the foci of their action, unless, indeed, their diffusion be promoted by the agency of the wind, or by direct transport. This subject, however, we cannot enter upon at present.

As to the influence of the Geological character of the soil on the development and diffusion of diseases, the Plague, like other marsh-maladies, has been observed to shew a marked preference to districts of an argillaceous formation. Pulmonary consumption and follicular Enteritis are said to be most prevalent in chalky countries; Goitre and Cretinism too have been alleged to be most common upon such formations; and some go so far as to tell us that it is from this circumstance that the latter name is derived."

"The character and composition of the water in any place should always be taken into account in studying its prevailing diseases. The water at Oran in Algeria is found to contain nearly twenty times as much matter dissolved or suspended in it, as that of the Seine. Surely such a circumstance as this cannot fail to have a decided influence on the health of the inhabitants: may it not account for the great frequency of dysentery in the former place?

M. *Boudin*, having discussed the various topics now alluded to, proceeds to examine the curious subject of the latency or *incubation* of many diseases—which may thus break forth at a distance from the point where their germs were developed. The period of concealment or abeyance, so to speak, of morbific poisons, within the human constitution, varies a great deal in different cases. That of Hydrophobia it is well known, may extend to three, six or even twelve months; that of marsh miasma for the same or even a longer period of time; and the malignant fever of Marseilles has been observed, in many instances, to be lying dormant for several months after the exposure of the person to the exciting causes.

The last Chapter of the work is occupied with a consideration of the Laws of Geographic Coincidence and Antagonism; more especially in reference to the alleged reciprocal repulsion of phthisis and typhoid fever on the one hand, and of marsh fevers on the other. This subject has of late excited very great attention in France, and has been repeatedly canvassed in the Royal Academy and other medical societies. M. *Boudin* is disposed to admit the correctness of the idea in question, and he has taken much trouble to shew, by an elaborate appeal to statistical reports, the extreme rarity of pulmonary Consumption and Typhus in all districts that suffer much from remittent and intermittent fevers. The long-established celebrity of d'Hyeres has been supposed to be owing, in a great measure, to the marshy character of the surrounding country.—*Gazette Medicale.*

*Remarks.*—While reading the preceding observations, more especially those which refer to the influence of the water, soil, &c. upon the diseases most prevalent in any district, we were strongly reminded of the admirable instructions given on this very subject by *Hippocrates* in his treatise " De aere, aquis et locis." Great stress is laid upon the necessity of paying due attention not only to the times and seasons of the year, but also to the situation and locality of any particular city or place; its exposure to the East or West, or to certain winds, &c.; the character of the surrounding country, whether this be marshy or dry, high or low; the quality of the water that is used as drink, and the general diet of the inhabitants; &c. The old Coan was much wiser in his generation than many of our modern *philosophes,* who, in their vain attempts to raise medicine to the rank of an exact science, seek to lay down certain fixed rules or principles to determine the treatment of diseases in all seasons and places alike.—(*Rev.*)

---

### Dr. Pereyra on the Treatment of Phthisis: Use of Cod-Liver Oil.

The author of this *brochure* has been one of the physicians of the St. Andrew Hospital, at Bourdeaux, for the last eight or ten years, and has had his attention particularly directed to the pathology and treatment of pulmonary Consumption. This sad scourge of France, as well as of our own country, seems to be exceedingly common in that city and its neighbourhood, in spite of the prevalence of ague and other marsh diseases, which arise from the damp character of the soil.

The chief object of Dr. *Pereyra*'s pamphlet is to recommend, to the notice of his professional brethren, the use of Cod-liver oil in the advanced stages of Phthisis, as a remedy that promises unquestionable benefit in many cases which have resisted every other known medicine. It is but fair to the author to state that, judging from his writing, he seems to be not only a practised stethoscopist, but also an intelligent and observing physician. There is no air of quackery about his statements; they are uniformly fair and candid.

In several of the cases related by him, in which a very remarkable suspension of the morbid degeneration of the pulmonary parenchyma took place, there were present all the symptoms (auscultatory as well as natural) of one or more tuberculous caverns in the upper lobes of the lungs.

The plan of treatment usually pursued was to exhibit the oil in doses of a table-spoonful or so, twice or thrice a day, and to put the patients on a nourishing and tonic course of diet.

Dr. P., also, in most of his cases has recourse to some artificial drain, established near the seat of the chief lesion—either on the parietes of the chest, or in the flesh of the shoulder. This is unquestionably good practice and deserves to be more generally adopted than it is.

Here is a brief account of one of the cases. A young prostitute was admitted into the hospital with all the symptoms of confirmed phthisis—hectic fever, night-sweats, purulent sputa, pectoriloquy, accompanied with a cavernous *souffle,* in two places over the upper part of the right side of the chest.

Under the treatment which we have menioned, the girl recovered her health astonishingly, and. after the lapse of three months, was so well as to leave the hospital. "I auscultated the chest at that time, and ascertained distinctly the existence of the two caverns; the crude tubercles seemed to me to be less extensive than they were some time ago, and over several parts of the affected lung the respiratory murmur was much more natural." Three years after this date, Dr. P. had the opportunity of repeating his examination, and then found that the pectoriloquy was still very audible over the same points that it had been—but the caverns now seemed to be decidedly smaller in their extent; and, as there was no longer any tuberculous *bruit* heard during respiration, he inferred that the surrounding substance of the lungs had partially recovered a more healthy condition.

It is quite unnecessary to adduce the particulars of more of the cases. In several of them, the accuracy of the diagnosis was entirely confirmed by the appearances found on dissection. This fact naturally gives us more confidence in the other statements of the author.

Even in the more successful cases, he distinctly warns his readers not to suppose that a complete cure, or restoration to a perfect state of health, took place, if there was any cavernous excavation in the substance of the lungs.

The excavation still continued, but the progress of disorganisation seemed to be arrested, and the indurated state of the surrounding pulmonary parenchyma was sensibly diminished.

The auscultatory phenomena shewed that such was the case; for the pectoriloquy and cavernous blowing were still audible, but the respiratory murmur was more uniform and regular.

One great drawback to the use of the cod-liver oil is its extreme nauseousness. Most patients vomit every dose for the first two or three days; but then, if steadily persevered with, it will usually remain on the stomach: it is curious, that young children will often swallow it without much repugnance. When the cough is very troublesome, Dr. P. recommends that the cyanuret of potassium be administered along with the oil: he has seen admirable effects from the judicious exhibition of this medicine. A nourishing diet of animal food, &c. is a powerful auxiliary to the use of the cod-liver oil, in enabling the system to stand up against the enfeebling effects of the disease, and to make an effort to arrest the disorganising process.

This treatment appears to be, on the whole, very judicious. Like every wise practitioner, Dr. P. varies it, as a matter of course, according to the character of the existing symptoms. If, for example, any signs of pleuritic complication come on during the use of the tonic regimen, he at once discontinues it, and has recourse to leeches, cupping, &c.—to resume it, however, when the inflammatory affection subsides.

He is a great friend to the use of counter-irritants, especially of issues and setons. He says: "In almost all my patients I establish a drain in the arm: this usually exerts a very favourable action upon the entire system. I always advise them to keep it open for several months after leaving the hospital, as it assists most powerfully in confirming the salutary check that may have been made on the disease. Those, who have had the discharge too soon dried up, speedily found that their cough, &c. became more troublesome; and many have therefore come back to have it re-established."

If diarrhœa be present, the cod-liver oil must usually be discontinued for a short time, and the strong diet be changed to one of a milder nature—such as rice, eggs, &c. The acetate of lead is one of the best remedies for internal exhibition under such circumstances; it serves also to check the night-sweats. Whenever the patient has a decidedly chlorotic look, Dr. P. recommends that steel medicines be freely given.

The following remarks by our author on the frequent coincidence of ague and consumption at Bourdeaux, deserve especial notice at the present time, when his countrymen make this question quite the "cheval de bataille" of their academic controversies.

"I must here," says he, "mention a complication which is perhaps peculiar to our geographical position; viz. a quotidian intermittent febrile condition, which has some points of resemblance with the paroxysms of the hectic fever, that is usually present in all cases of confirmed phthisis. But they may be distinguished by the circumstance of the attacks of the former being always more regular and stated than those of the latter. The shivering of ague usually occurs about the middle of the day, and the strong sweating stage about midnight. Whenever we have reason to suspect the existence of a co-existent ague, we should have recourse to the quinine, without discontinuing the use of the former medicines and regimen. Usually the patients bear the quinime remarkably well, and its effects are so speedy and decided that, after the lapse of a week or so, we are enabled to dispense with it. * * *

"Intermittent fevers are so prevalent in and around Bourdeaux, that they constitute more than a fourth part of our diseases. Their presence in the system decidedly promotes and accelerates any tendency that there may be to tuberculous deposits.

"As we have already explained, whenever we have reason to suspect the existence of such a complication, we should at once get rid of the ague by the administration of the quinine, either alone or in conjunction with opium."

Dr. P. mentions it, as the result of wide observation, that "one of the most frequent causes of phthisis among the youths in Bourdeaux is the depraved habit of masturbation. There are few young men, who have not confessed it to me; and I have strong reason to believe that those of the other sex, although less willing to acknowledge their fault, are quite as culpable."

The question as to the communicability of consumption from one person to another has usually been decided in the negative. Our author however intimates his doubts upon the subject; having seen so many cases in which the disease appeared to have been transmitted to persons who have been in constant attendance upon the sick. Often has he observed strong robust women begin to exhibit phthisical symptoms not long after the death of their husbands from the disease; and occasionally also the reverse of this. It is therefore only a wise precaution to avoid, as much as possible, inhaling the breath of any one labouring under the advanced stage of consumption. Dr. P. alludes also to the well-known circumstance of many famous chest-doctors—for example, *Laennec, Bayle, Delaberge, Dance,* &c.—having fallen a sacrifice to the disease, and thinks it not at all unreasonable to suppose that their continual and often very prolonged visitation of phthisical patients may have had something to do with the origin of their ailments. He therefore strongly advises those physicians and students, who may have any hereditary tendency to the disease, to be more than usually cautious not to inhale the breath of such patients, and to avoid unnecessary delay in their visits.—*Du Traitement de la Phthisie Pulmonaire, par E. Pereyra,* pp. 84. 1843.

*Remarks.*—That so penetrating and active a medicine as cod-liver oil—which unquestionably possesses, according to all accounts, no ordinary remedial powers in many forms of scrofulous disease—may be of decided benefit in certain cases of pulmonary phthisis, is far from being unreasonable. It appears to be a general stimulant, in a moderate degree, of every part of the system, and, from the small portion of iodine present in its composition, it is well suited, we should think, for persons of a strumous habit. Whether a due combination of this potent substance with some of the vegetable balsams, such as the Peruvian or the copaiba, might not be substituted for the cod-oil—either when this cannot be procured, or when the patient's stomach will not retain it—deserves a trial. We know that all the medicines of this class (the balsamic) have a marked influence on the respiration; they appear to be very rapidly absorbed, and conveyed out of the system, partly by the lungs and partly by the kidneys. In many cases of chronic bronchitis, senile catarrh, peripneumonia notha, &c., no remedy is better than the Copaiba or Peruvian balsam. Is it not possible that, when iodine is administered in combination with such substances, it may be directed in an especial manner to those organs which they more immediately affect?

The practice, adopted and recommended by Dr. *Pereyra*, of establishing an artificial drain in the immediate neighbourhood of the chest, (as in the flesh of the shoulder,) whenever one of the upper lobes of the lungs is diseased, is unquestionably very judicious, and should be followed more generally than it is. We have often witnessed decidedly good effects from this plan, in retarding the progress of the pulmonary lesion, and can therefore bear witness to the wisdom of the advice. The use too of a nourishing, but not a stimulating, diet, so as to support without over-exciting the powers of the system, should never be neglected, whenever the patient's stomach will bear it. On the whole, we report favourably of this pamphlet of Dr. *Pereyra*: it is a candid and faithful statement of an enlightened physician.—*Rev.*

---

### PHYSIOLOGICAL MEMORANDA ON THE RELATIONS BETWEEN DIGESTION AND RESPIRATION, &c.

The following observations appear to be based on those interesting researches, which of late years have been so assiduously carried out by MM. *Dumas* and *Boussingault* in France, and by M. *Liebig* and others in Germany. The great interest and importance of the subject will always command for it the attention of those who take a pleasure in making themselves acquainted with the marvellous workings of the living system—as they are revealed in the unmarred book of Nature herself, and not in the disgusting exhibitions of a vivisection room.

"The activity of the Digestion appears to be, in a great measure, influenced by, and proportionate to, that of the Respiration. Whenever the latter function is very highly developed, or more than usually vigorous, we find that the animal will require food more frequently and in larger quantities than under the opposite conditions. A bird will die from starvation in the course of two or three days; while a serpent will live for months without any supply of nourishment. For the same reason, an infant, whose respiration is well-known to be much more rapid than that of an adult, requires to be fed more frequently for the first year than in after-life. How is it that hybernating animals can survive a whole Winter in their state of almost asphyxiated inaction?—simply because the respiratory process is all but arrested. During ordinary sleep the breathing is much slower than when we are awake and taking exercise; and hence it is (in some degree at least) that no desire for food is experienced for so many hours.—Every one must have found by his own experience that lively conversation after eating is one of the best digesters. May not this be owing in part to the active play of the respiration, as well as to the general pleasing excitement of the whole nervous system?"

"In estimating the activity of the breathing, (and consequently the quantity of oxygen absorbed into, and of carbon eliminated from, the animal system), we must attend not only to the number of respirations in a given space of time, but also to the temperature of the air that is respired. As the same, or nearly the same, volume of air is inhaled at each act of breathing in all seasons, it is quite obvious that, unless the respiration be considerably quicker in Summer than in Winter, the consumption of oxygen must be much less at one season than at another, and therefore (as explained in the preceding paragraph) that the necessity for food will become so much the less urgent. Now, although the breathing is somewhat quicker in warm than in cold weather, the increased frequency is by no means proportionate to the diminished density of the air. Hence it may justly be laid down as a general truth that an animal consumes less oxygen in Summer than in Winter, and therefore that it requires a smaller amount of nourishment. Does not the experience of every man prove the truth of this observation in his own case? and is it not the case that his appetite for strong animal food is much greater, when the weather is cold and bracing than when it is hot and relaxing? We must not, it is true, omit from our consideration the direct influence of various outward impressions on the Nervous system; the digestion, like every other function of a living body, is not a little affected by whatever either invigorates or enfeebles the general powers of the constitution. But, independently of this *vital* or *animal* agency, there is another of a *chemical* and *organic* kind, (such as we have been endeavouring to explain) which has never been rightly appreciated until of late years, when Animal and Vegetable Chemistry has been studied with such a philosophic spirit. By the act of Respiration, a certain quantity of oxygen is absorbed into, and a certain quantity of carbonic acid is eliminated from, a living animal body. As the basis of the latter gaseous substance must unquestionably be derived primarily from the food that is taken into the system, it may at once be presumed *à priori* that there is a direct relation and sympathy between these two most important functions."

"If then the consumption of oxygen during the process of respiration is so much more active in cold than in warm weather, it may naturally be asked how is it that the system relieves itself of the excess? or in what manner does it employ the large supply of this element in the first of these conditions? The answer to this is easy. For in proportion to the amount of oxygen absorbed by, so is that of carbonic acid evolved from, the lungs. Now the amount of the carbonic acid evolved depends entirely on the activity of the digestion, and on the quantity of carbon which is derived from the food that is eaten, and which becomes incorporated with the blood. When the digestion is vigorous, and the quantity of food consumed is great, a large supply of materials abounding in carbon and other elements is continually being added to the mass of the circulating fluid. These materials serve for different purposes. Besides contributing to the formation of new compounds to repair the constant waste that is going on in every part, a large proportion of the carbon combines with the oxygen that is always present in the blood, and passes out of the system at every act of respiration. The lungs are therefore the great emunctories for the discharge of carbon out of the body. * * * * *

"Such being the case, we can at once understand the reason why the system requires a larger supply of strong nutritious food in Winter than in Summer, and why the inhabitants of the Arctic regions live almost exclusively upon articles of an animal nature, while those of the tropics may be sustained by a diet that consists almost entirely of vegetable matters. In the former, a much larger amount of oxygen is consumed during respiration, in consequence of the greater density of the air that is respired. The result of this is that the digestive function is exceedingly active, and it therefore requires a greater supply of strong food, in order to furnish a sufficient quantity of carbon, &c. for the formation of the large supply of carbonic acid, that is afterwards eliminated by the lungs.

"For the reasons now given, it may be readily understood why it is so much easier to fast for a length of time in warm than in cold weather, and why a lighter diet should be used in Summer than in Winter. Hence too we perceive the cause that an ill-fed animal will become emaciated much more rapidly in the latter than in the former season; for, if there be not a sufficient supply of carbon yielded by the food in order to combine with the oxygen absorbed during respiration, the system must derive it somehow, and this will be at the expence of the substance of its own body."

"The reciprocal action of the elements of the food on the one hand, and of the oxygen diffused through every part of the body, by means of the circulation, on the other, is the source of Animal Heat. This most important and curious function is now recognised to be owing (at least, in a very great measure) to the direct union of the oxygen in the blood with some combustible substance of a carbonaceous nature. It is obvious therefore that, just in proportion to the amount of carbonic acid that is formed within the system and evolved during respiration, so will be the degree of caloric generated in the animal system. Now we have already seen that this amount is influenced by the activity of the respiratory changes on the one hand, and by the quantity and quality of the food that is consumed on the other. Whenever the respiration is quick and active, the heat of the animal body is high; and *vice versâ*. For this reason it is that the temperature of birds is higher than that of any other class of animals, and that the young creature is always so much warmer than the old. In common parlance we say that in age the blood is *chilled:* the remark is quite true, physically as well as poetically.

"Again, we have strong grounds for believing that more caloric is generated in the animal body during cold than during hot weather. Indeed, this must be so; for, as there is a larger absorption of oxygen and a larger formation of carbonic acid in the one condition than in the other, so there must necessarily be a greater development of caloric produced. It is thus that Nature so admirably compensates for the great abstraction of heat from an animal body during Winter, when the temperature of the air is so much below that of its surface. It is only on some principle like this, that we can account for the wonderful uniformity of the temperature of living animals of the same kind in different latitudes:—less heat being generated within the body in hot climates, because less is abstracted from it; and *vice versâ*. As we have already explained, the cause of the lesser generation of animal heat in the one case than in the other is the lesser formation of carbonic acid within the body; and this again is in consequence, on the one hand, of the smaller consumption of oxygen during the process of respiration, and on the other, of the smaller amount of carbon received from the food that is eaten. The fruits, which serve as the chief means of nourishment to the inhabitants of tropical countries, do not contain above 12 per cent. of carbon; while the whale blubber and seal oil, on which the Laplander feeds, contain as much as from 65 to 80 per cent. of the same material. In Winter we all find the need of an animal diet; in Summer we require but little flesh food, and instinctively long for a lighter diet of vegetables and fruits. We thus perceive how beautifully Nature has accommodated all her arrangements to the varying conditions of season and climate; and we, at the same time, derive an important dietetic rule for the regulation of our living, according to the circumstances in which we are placed."

"From what has been said, in some of the preceding paragraphs, it will be obvious to every one that there is, and must necessarily be, an intimate relation between the activity of the Respiration and the consumption of oxygen during this process, on the one hand, and the activity of the Digestion and the introduction of carbonaceous and other matters into the blood, on the other. Unless this relation be harmonious and equable, the state of the health will quickly proclaim that there is some disturbance in the balancing powers of the animal economy. If a sufficient supply of carbonaceous materials be not provided by the digestive process, while the consumption of oxygen in the lungs is large, there will be rapid emaciation of the body—in consequence of the absorption of every particle of fatty matter that it contains, to supply the demand for carbon that the absorbed oxygen is continually making for the formation of Carbonic Acid.

"On the other hand, if the supply of carbon furnished by the digestive function to the blood exceeds the proportion that is required to satisfy the demands of the oxygen consumed, then it will either accumulate under some form or another within the body, or it must be discharged from the system by some other way than by that of respiration.

"In the one case, there will be a deposit of fat in the cellular tissue; and in the other, an extra duty, so to speak, will be thrown upon the liver, and this organ becomes stimulated to secrete a larger quantity of bile than is necessary in a state of perfect health.

"It has been long known that the Biliary secretion abounds in carbonaceous matter; indeed many physiologists have regarded the liver as the great eliminator of carbon from the system. The fact is true, although the theory may not be quite correct. Certain however it is that, whenever the carbon in the system is not duly eliminated by the lungs, there is usually forthwith an increased action of the liver set up; and *vice versâ*. These two organs therefore, appear to act as balancing or compensation agents in the important process of discharging the carbonaceous matter out of the system; and thus it is that, when there is an undue amount of duty, so to speak, thrown upon one of these organs, the over-acting viscus becomes more than usually subject to disease. In hot climates it is the liver, and in cold climates it is the lungs, that most frequently suffers. Are we not thus, by a very simple train of observation, led to perceive the importance of suiting the diet to the climate in which we reside? The practice of so many of the English, in the East and West Indies, of eating as much animal food under a tropical sun and in an attenuated atmosphere, as they were in the habit of doing in their own country, is well known to be a prolific cause of disease. A little attention to the simple suggestions of Nature herself—quite independently of the researches of the chemist and physiologist as to the *why* and the wherefore—might annually save many valuable lives."

"The refrigeration of the body, from whatever cause this may proceed, is generally found to increase the desire for food. Mere exposure to the air in an open carriage or on the deck of a ship, although no active exercise be taken, whets and invigorates the appetite. With many people a large draught of cold water will produce a like effect, and with others the exercise of the lungs in singing, speaking aloud, &c. will do the same. In what manner does the impression of cold on the body produce an increase of appetite? Its action seems to be two-fold: in the first place, it tends to brace and stimulate the whole body, more especially the muscular and nervous systems; and, 2ndly, by carrying off the caloric of the body quickly, it no doubt promotes the combination of the oxygen absorbed by the lungs with the carbon existing in the blood. If then this combination take place more rapidly than usual, there will be a greater demand for a supply of fresh pabulum to compensate for the increased expenditure."

"No one can reasonably dispute the influence of the nervous system on the function of Respiration, as well as on the muscular powers that move the chest. Without the agency of the nerves, the stomach and intestines cannot prepare the elements, that are destined to combine with the oxygen absorbed during every act of breathing; and thus it is that this very act of absorption itself must also speedily cease. If the *pons Varoli* in a living animal be cut, or if it be stunned by a blow on the top of the head, it will continue to breathe for some time—indeed the breathing may be more rapid than it was before;—but the heat of the body rapidly becomes less and less, until life is extinct. Now what is the cause of this? It seems to be that, as the absorbed oxygen no longer meets with a due proportion of the substances (carbon and hydrogen) with which it is destined to combine in the blood, the combustion, so to speak, is arrested; and thus there is no longer any fresh generation of animal heat.............. As the section of the pneumo-gastric nerves causes a cessation of the contractions of the stomach, and of the secretion of the gastric juice, and thus puts an immediate stop to the process of Digestion, in like manner any palsy or excessive weakness of the nerves of the bowels is invariably followed by some disturbance in the act of Respiration. Those two functions are united together by the closest ties of sympathy; and therefore we cannot be surprised that a lesion of one function should be almost invariably followed by a lesion of the other."

"Admitting that the existence of Magnetic and Electrical currents in the animal body may have some share in the development of the functions of its different organs, the ultimate cause or generating power of all these agencies is the transition of matter from one state of existence to another, during the process of transformation of the elements of the food into oxygenised compounds: viz. carbonic acid and water—those, which do not undergo this process of slow and gradual combustion, being rejected from the body under the form of excrementitious matters.............. Now it is quite impossible that any given quantity of carbon or of hydrogen—whatever be the forms which they assume during this act of combination—can generate more caloric within an animal body, than is produced by the direct combustion of these elements in oxygen, or in atmospheric air. It is not indeed easy to determine with accuracy the amount of carbonic acid that is evolved from the lungs, or of the watery vapour that is exhaled not only from them, but also from the surface of the body. But perfect accuracy on this point could not assist us very materially in our physiological enquiries; nor does the want of it invalidate the general correctness of the conclusions which we have been endeavouring to establish."—*Annalen der Chemie, &c.*

---

### M. RACIBORSKI ON MENSTRUATION.

This very intelligent and active physician of the French metropolis has, within the last few months, read a very elaborate memoir on this much discussed subject before the Royal Academy of Medicine. Like most French medical writings, this memoir is far too lengthy and circumstantial; a vast number of particulars of little or no interest, or which are well known to every tyro in the profession, being most unnecessarily introduced. The following summary, however, of the author's views will well repay the trouble of a perusal.

According to M. *Raciborski*, the Menstrual discharge is (we use his own words) "an epiphenomenon (accessory phenomenon) of an important function devolved upon the ovaries, viz: the successive formation and separation of the ova and Graafian vesicles." It is not therefore, as many physiologists imagine, a mere issue or secretion intended to relieve the system of an over-supply of blood in the unimpregnated state; nor yet, as has been supposed by others, an important crisis which Nature often employs to abridge the duration of various diseases, and the least derangement of which is apt to be followed by a constitutional disturbance.

Formed in the very first year of life, or even sometimes before the birth of the infant, the Graafian follicles are found to go on gradually increasing in size and number, until they ultimately arrive at their complete development. The period of life at which this takes place, varies a good deal according to the vigour of the system, and the general hygienic conditions to which the body is exposed during the early years of life: it coincides in the human female with the appearance of the usual signs of puberty and with the first show of the catamenial discharge. Hence, whatever accelerates or retards the maturation of the Graafian follicles, is observed to accelerate or retard the phenomena in question.

Many medical men—erroneously attributing the many complaints, to which some girls are subject at the epoch of puberty, to the mere efforts of the system to bring on menstruation,—endeavour by a variety of means to cause a determination of blood to the sexual organs, with the view, as they imagine, to assist nature. The true cause of the very common unsuccess of such endeavours is what we have mentioned above, viz.: that the Catamenial secretion is not a mere independent discharge of blood to serve any particular purpose, but is in fact the result and consequence of a peculiar process which has gone before it.

Whenever the development of the Graafian follicles is not retarded by any latent cachectic condition of the system, they gradually increase in number and dimensions, while at the same time they approach nearer to the surface of the ovaries. About the period of puberty, we may sometimes count as many as between 30 and 40 in each ovary, and often not a few of them are distinctly visible through the external envelope of this organ.

From the researches of MM. *Coste, Carus, Valentin, Wagner* and others, it appears that the germ in the human female (as in the females of the lower animals) consists in the presence of an ovum, similar to what is found in the bird. This is a curious fact; for it shows that the law, which regulates the generation of animals, is alike in all. Now the sphere of this law, so to speak, will be most unexpectedly enlarged, if we can shew that, in the females of all mammiferous animals, just as those of birds, fish, reptiles, &c. there is a spontaneous periodic discharge of ova, without the intervention or co-operation of the male sex. Our researches, we think, clearly establish the truth of this curious position.

At each menstrual epoch, a Graafian follicle forms a projection on the surface of the ovary, then becomes the seat of a hæmorrhage, and finally bursts and gives issue to the ovum which it contained.

The catamenial discharge appears to be the result of the sanguineous congestion in the generative organs, which accompanies the last stage of the development of the follicles.

Whenever we have occasion to examine the body of a female who has died very soon after the catamenia have been upon her, we may feel confident that we shall find traces in the ovaries of a 'ponte' (laying of eggs), that has quite recently taken place.

Although M. *Negrier* had clearly foreseen that such must be the case, he was not able, for want of favourable cases, to demonstrate the perfect truth of this fact. None of the observations, which this eminent physician has published, affords any decisive refutation of the old opinion held by *Haller, Graaf,* &c., and which is still received in the schools,—viz. that a rupture of the follicles never takes place except during or after coition.

The females of all mammiferous animals are subject to a similar 'ponte,' during the periods of rutting or heat. One or more of the follicles, according as the animal is uniparous or multiparous, then attain their maximum of developement, and form a projection on the surface of the ovary. At length they become the seat of a hæmorrhage, are filled with blood, and finally burst and give issue to an ovum. These phenomena are best observed in the sow, in consequence of the position of its follicles, which exhibit the appearance, in some respects, of a cluster of grapes. By attentively examining the ovaries in this animal, we may easily form an idea of what becomes of the follicles, after the discharge of the ova, and how they shrink to such small dimensions soon afterwards, as to be only indistinctly visible—no trace, perhaps, remaining except a minute yellowish or orange-coloured speck.

The escape of ova takes place spontaneously in the lower animals, as it does in the human female, without any intervention of the male. We have ascertained the perfect accuracy of this assertion in swine and in bitches, which had been kept apart from their mates during the whole period of rutting.

It is but justice to admit that *Vallisnieri, Malpighi,* and *Bertrandi* had, many years ago, remarked the spontaneous rupture of certain parts of the ovaries in different animals during this period. These physiologists, however, fell into the error of supposing that these bodies, which they called by the name of *corpora lutea,* or *corpora glandulosa,* were really distinct organs destined to bring the ova forward to maturation. Now it may be unhesitatingly maintained, that all that has at any time been said of the so-called *corpora lutea,* &c. is exclusively applicable to the Graafian follicles at a tolerably advanced stage of their formation.

Whether the rupture of the follicles and the subsequent escape of the ova take place spontaneously, or be the result of copulation, the appearances which they (the follicles) exhibit afterwards, are very much alike: this remark applies to the female of the human subject, as well as of the lower mammiferous animals. Indeed, there seem to be certain cases, where the venereal orgasm alone, during coition, is accompanied with a rupture of the follicles, without any previous preparation on the part of nature; but then, before the 'ponte' can take place, the follicles must pass through all the degrees, so to speak, which they usually undergo about the period of the spontaneous expulsion of the ova. The external envelope of the ovary is swollen, becomes the seat of a hæmorrhage, and afterwards the escape of the ovum takes place. We may observe this phenomenon in rabbits, which copulate at all seasons and do not wait for any particular period.

It thus appears that women, like the females of all the mammiferous animals, are subject to a spontaneous 'ponte,' which recurs at particular epochs—known in the case of the former as the menstrual, and in that of the latter as the rutting, periods. The process in question is evidently connected with the reproduction of the species. In the majority of animals, there cannot be a doubt on this point; for there are some females which will not allow the approach of the male, until they are prepared by nature for the 'ponte.' The human female must be placed intermediate between such animals as are subject to periodic rutting, and those which are capable of generating at all seasons. On the whole, however, she approaches more nearly to the first, than to the second, of these classes.

One result from our statistical researches—which were made with the greatest exactitude—is that, out of 100 pregnant women, not more than six or seven at most became so by sexual connexion at a tolerably distant period after menstruation. In most women, conception may be dated from either a few days before, or a few days after, the menstrual period.

It is well known that mule animals, female as well as male, are incapable of reproduction. What is the cause of this sterility? Most probably the absence of Graafian follicles in the ovaries of the former, and of spermatic animalculæ in the seminal fluid of the latter.—*L'Experience.*

---

### ON THE ORIGIN OF MILITARY OPHTHALMIA.

A report upon the very instructive researches of Dr. *Caffe* on the Ophthalmia of armies, and especially on that which is still prevalent throughout Belgium, was recently read before the Historic Institute of France. The following extracts will be read with some interest.

"In examining the question, as to the origin of the ophthalmia in the Belgian army, it deserves especial notice that the disease was not known in it until the year 1814, when it made its appearance among the troops of the 7th battalion, posted at Gand. There cannot be a doubt on this point. Well then, the disease must either have been of indigenous development, or it must have been imported somehow into the country. All testimony is against the first, and in favour of the second, of these suppositions. The old soldiers, who formed, as it were, the nucleus of this battalion, had contracted the disease while they served in the French army. It was from them that it was communicated to the young recruits.

"Admitting, then, the truth of the fact, that the Belgian army derived the disease from the French army, how shall we explain its first introduction into the latter? All the best authorities trace this to the memorable campaign of our troops in Egypt. This country—which seems to have the dismal prerogative of engendering most of the pestilences which have desolated the world—has an atmosphere so loaded with saline particles * that we cannot wonder much that the eyes of the inhabitants are almost continually suffering; more especially when, to this source of irritation, we have to add the dazzling reflection of the sun's rays from the plains of sand everywhere—the true cause, by-the-bye, of that singular phenomenon, the *mirage*.

"Then, too, the winds from the desert—so well known by the poetic appellations of Simoom and Samiel—are so utterly desiccative, that the ground becomes dry almost the very moment after it has been watered, and all vegetation is quickly withered up.

"While these winds blow, the skin feels quite harsh and crisped up, and the whole system is in a state of feverish excitement; the eye, as a matter of course, must suffer in an especial degree. We cannot, therefore, be surprised at the extreme frequency and severity of Ophthalmia in Egypt. The constantly increasing number of miserably blind creatures in that country forms one of the greatest drawbacks to its national prosperity.

"This seems, indeed, to have been the case from the earliest times. *Herodotus* tells us that *Cyrus* sent a deputation into Egypt to obtain the assistance of a skilful oculist; and we read that *Cambyses'* army, after crossing one of the sandy deserts, returned to their own country in the most miserable plight, in consequence of so many of the poor soldiers having become blind and otherwise diseased. Many of the troops in *Alexander*'s army, that invaded Egypt, suffered terribly in their eyes; and so great was the discontent at one time among his soldiers, that there was every prospect of a general mutiny. The Roman armies, too, had a great aversion to military service in this part of the world. In early French history, we meet with several notices to the same effect. During the reign of Louis IX. many soldiers, who had accompanied an expedition into Egypt, returned either quite blind, or suffering with diseased eyes. It is scarcely necessary to allude to the results, in this respect, of our last expedition under the modern Alexander—who has not however left a city behind him, to proclaim the glory of his name. But as no epidemic ophthalmia had been known in the French army from the time of St. Louis down to the close of last century, the disease, when it was brought back by Buonaparte's troops to France, was regarded as quite novel and unknown in medical history. So severely did our

---

* "The salt is so abundant, that every pit that is dug becomes filled with a saltish water: this is found to be owing to the presence of marine salt, natron, and a little nitre. The surface of the ground, that has been well watered, is often observed to be quite crusted over with a deposit of salt. The natron is formed in great abundance in the different lakes, and large quantities are collected along the track of the Isthmus of Suez."

poor soldiers suffer, that in Desaix's corps, at El. Laoum, there were not fewer than 800 threatened with complete blindness. During the whole period of occupation, the army suffered terribly from the epidemic. Every now and then it would seem to be disappearing; but again and again it broke forth with as much severity as ever. At length it yielded (or seemed to do so) to an epidemic dysentery;—the irritation of the bowels acting as a potent revulsive to that of the organs of vision.

"Let us briefly follow the course of our troops from the time when they left Egypt, in October 1801, until they landed at Toulon and Marseilles.

"Of the whole French fleet, which sailed for Egypt under Admiral *Brueis*, only two line-of-battle ships (the Guillaume Tell and the Genereux), and two frigates (the Justice and the Diana) escaped after the battle of Aboukir. These vessels, under the command of Admiral *Villeneuve*, reached Malta. A part of the fleet was burned, destroyed, or carried as prizes to Sicily by *Nelson*. A number of smaller craft remained at a distance from the scene of action, and escaped at first; but most of them were afterwards captured by the English. One vessel, with 150 wounded and blind on board, reached the shore of Sicily; but the whole crew was barbarously murdered by the inhabitants. The transport, that had *Junot*, *Rigel*, *Lallemand*, &c. on board, was taken by the enemy's cruisers; while that, in which many of the other chief officers had embarked, was driven on shore in the Gulf of Tarentum. In short, with the exception of the squadron which, under the command of *Gantheaume*, eluded the vigilance of the British and at length landed Napoleon at Frejus in 1800, and of the Lodi which carried General Leclerc to St. Domingo, nothing remained of that fine fleet that, two years before, had carried Cæsar and his fortunes to the East. The rest of our navy subsequently disappeared at Trafalgar in 1805, and at Cadiz in 1808.

"It is evident, therefore, that it was not the French fleet which could have imported the Egyptian ophthalmia; seeing that the fleet itself was destroyed in the East, and its poor remnant went to the West Indies, there to perish of the yellow fever. (The justness of this conclusion is not at all so obvious to us as it seems to be to the writer). Let us now follow the course of the troops, and see whether we can thus obtain any light upon the subject.

"Just before the capitulation of the army, the Ophthalmia (as already mentioned), which had shortly before broke out with great severity, had begun to abate under the influence of a scorbutic dysentery. Still there were very many of the soldiers grievously affected with it, when they embarked on board the British flotilla. Ultimately they were all landed at Marseilles. The troops, which a twelvemonth before had arrived with Buonaparte at Frejus, had unquestionably first brought over the germs of the disease, as a good many of the men (belonging to the small division that was known as the Hussars of Argento) were suffering from it at the time of their debarkation. Part of these were incorporated with the corps of the Mamelukes, and with them stationed at Melun—where the disease was not long of breaking out with violence among the young recruits. About the same time, the troops that had formed the garrison of Cairo, under the command of Belliard, returned to France in British transports; and *Samuel Cooper* tells us that, happening to be at Marseilles in the year 1802, he himself saw many of the French soldiers affected with the ophthalmia, which they had brought with them from Egypt.

"It was by the sick on board the first English transport after the capitulation of Alexandria, and by the hussars of Argento, and the garrison of Cairo—these brave fellows becoming gradually dispersed over almost every part of Europe—that the infection was introduced into France, Italy, Holland, Germany, and Belgium. The latter country alone retains in the present day this ill-starred gift of the conquerors of the Pyramids to the Grand Army. Such is the origin of the Belgian ophthalmia—a living branch, so to speak, of a poison tree that grows in the plains of Egypt."

At the same time we must admit that it is far from easy to explain satisfactorily why the disease should of late years have been confined to one country only of Europe, while others, that were originally infected, are now quite exempt from it. How far the following explanation will suffice, may seem doubtful; but it deserves notice. "If we except the Mamelukes, who formed a distinct corps to the close of the Empire, all the regiments of the Egyptian army were dissolved, and their men incorporated with other regiments that eventually became dispersed over almost every country in Europe. Engaged in incessant marchings or constantly-renewed combats, one day encamped in one place and next day in another, they seldom remained for any considerable time in barracks. Now it has been proved that the frequent change of air, and not crowding the sick too much together, are two of the most efficient means to check the progress of military ophthalmia. The disease was thus restricted to the actually existing cases, and was scarcely at all communicated to others. In consequence of this, fresh cases were of rare occurrence, and the extent of the mischief was gradually becoming more and more contracted. As a matter of course, the greater number of the poor fellows, who were afflicted with it, were killed in some one of the murderous battles that were every now and then fought. Thus it was that, in 1814 and 1815, when France was over-run with hostile armies, not above a very few hundred cases were to be found in all the hospitals put together. The foreign soldiers in the French army being then sent back to their respective countries, it so happened that a battalion, that had been formed at Gand in 1814, received into its ranks a good many men that were affected at the time with ophthalmia. From this *focus*, at first of a very limited extent, there was gradually diffused the baleful evil, which has ultimately extended itself to the entire Belgian army.

"Such is the opinion which we have formed upon the subject; and we may therefore sum up our observations by saying that the ophthalmia of armies,—the offspring of that ophthalmia which from time immemorial has been known to prevail in Egypt, after afflicting every country in Europe (England not excepted)—has remained contagious and epidemic in Belgium alone, where a single exceptional circumstance has caused it to survive, while in every other country it has become extinct and unknown."—*L'Examinateur Medical.*

---

### M. Rayer on Typhus in the Lower Animals.

How far the following communication, from the pen of this eminent physician, may prove satisfactory to the majority of readers on this side of the Channel we shall not take upon ourselves just now to decide, but append a few remarks at the close of it. It was sent to the Royal Academy of Medicine in the form of a letter addressed to the secretary.

"For the last twenty years, the subject of *entero-mesenteric* or *typhoid* fever has excited the most lively attention among the medical men of all countries. We cannot be much surprised at this when we consider the very great frequency, and the, alas! too common danger, of the disease.

"The comparative study of man and of the lower animals naturally leads us to enquire if a malady, that is of such frequent occurrence in the former, is ever met with in the latter, and to ask ourselves whether the silence of veterinary writers on the subject is to be regarded as a sufficient warrant to negative this proposition, or merely as shewing that the intestinal lesion, which constitutes the most positive anatomical character of this disease, may have hitherto been overlooked in the case of the lower animals, just as it was in the human subject, before the researches of MM. *Petit* and *Serres* came to be generally known.

"I was in this state of uncertainty, when chance presented to my notice, a few days ago, a most favourable opportunity of witnessing a case which has had the effect of leaving no doubt on my mind that Entero-mesenteric Fever does actually occur in certain of the solipedous animals.

"A donkey, about six weeks old, died after having been affected with a severe diarrhœa for about eight days. It was brought to my laboratory; and on examining the body, I could find no traces of disease anywhere, save and except those very lesions which we meet with in a human being, that has died during the first stage of typhoid fever. I have now the honour to submit to the inspection of the Academy a faithful representation of the vascular injection observed in the small intestine, cæcum, and commencement of the colon.

"I may here mention that the Peyerian glands in this animal are naturally much developed; but their appearance is very different from what was found in the specimen that is now exhibited. For here not only do they project considerably upon the internal surface of the gut, but several of them present a decidedly red colour, while around others the mucous membrane itself is more or less highly congested. One of the groupes was ulcerated about its centre; but most of them were merely puffy and tumefied. The mucous lining of the small gut was of a reddish colour throughout: this appearance was most conspicuous in the jejunum and upper part of the ileum The entire length of this intestine was filled with a dirty-looking grey or reddish-coloured fluid. There was no appearance, in any part either of the small or large bowels, of the plastic lymph which is generally observed in cases of genuine dysentery. The mesenteric glands were swollen, and several of them were so highly injected with blood as to have a red colour; others had only a rosy hue, with numerous dark striæ on their surface. The anatomical appearances therefore in the ileum and mesenteric glands were clearly such as are observed in the bodies of those, who die in the first stage of typhoid fever. The same thing may be said of those presented by the cæcum; for the lining membrane of this gut also was of a lively red colour, and looked as if it were covered with a considerable eruption of papulæ, attributable to the increased development of the isolated crypts of this bowel. .. .. .. .. .. The pyloric extremity of the stomach exhibited a large patch of ecchymosis; the spleen, liver, kidneys, and bladder were sound; so likewise were the lungs, heart, brain, &c.

"We thus perceive that in this animal, which died of an acute disease chiefly characterised by the existence of diarrhœa, the only morbid appearances witnessed on dissection were an increased development of the Peyerian glands, ulceration of one groupe of them, a general enlargement of the solitary glands of the large intestine, redness of the mucous membrane of all the bowels, tumefaction of the mesenteric glands, and the presence of a sanguineous fluid in several parts of the small intestine—an *ensemble* of lesions, which, as far as we know, has not its analogue, except in those which characterise typhoid fever in man."

Such is the case adduced to prove the existence of typhus in the lower animals: now for the learned reflections of the accomplished narrator.

"I must however admit that the question, as to the existence of this fever in the lower animals, is not completely solved by a single fact; but the present instance may probably suffice to draw the attention of veterinary practitioners to the subject, and lead them to examine, more minutely than they have hitherto done, the state of the intestinal mucous glands in the serous and sanguinolent diarrhœa which is so common in the young of our domestic animals.

"The allusions to the state of these glands in veterinary writings are but few, and very far from satisfactory. In the numerous works, which have at different times been published on the *typhoid affections of cattle*, we find the authors insisting much on the diffused and ecchymotic redness of the intestines, the swelling and softened condition of the spleen, occasionally the softening of the lungs also, the alteration of the blood—morbid states which are tolerably common in certain forms of Typhoid fever. But little or no notice is made of any lesions of the intestinal and mesenteric glands—the very lesions to which so much importance is attached in human pathology."—*Gazette Medicale.*

*Remarks.*—It is certainly not from any importance in the contents of the preceding note that we are induced to append a few observations of our own, but rather from our surprise that so distinguished a physician as M. *Rayer* should have given expression to such statements as we find in it.

That a man of his experience and research should deem it worth his while to address a formal letter to the leading medical association in his country, in order that he might convey to its learned members the important intelligence that he had found the mucous and mesenteric glands somewhat red and swollen in a young ass which had been affected with diarrhœa, seems to us most passing strange! And then too the extraordinary inference which he draws from this interesting discovery:—what is it?—nothing more nor less than that this one fact affords a strong and very reasonable presumption that Typhoid fever may affect the lower animals as well as man!

Some of our readers may say that they do not perceive the *sequitur* in this important proposition. If so, we take the liberty of telling them that their blindness proceeds not so much from the darkness or uncertainty of the problem itself, as from the dulness of their own minds. Do they not know that it is an undisputed (at least by the majority of the French physicians) axiom in pathology, that typhoid fever is a certain morbid state of the intestinal and mesenteric glands? and is it not therefore as clear as noon-day that the converse of the axiom must also be true, viz. that this said morbid state of the bowels is typhoid fever? Let them observe for a moment how this simple syllogism explains and illustrates much that is otherwise obscure in pathology. For example, the blood is well known to be fluid after death from lightning; so it is also in many cases of fatal poisoning; as well as in putrid fevers; in pestilential cholera, &c. Here then is a feature common to all these morbid states. Is not the inference then quite transparent, that they are all identical in their nature, or at least that they all belong to the same family? Such we think to be a fair parallel to the case adduced by M. *Rayer,* and the reasoning which he has founded upon it.

That the mucous membrane and glands of the intestines should exhibit some traces of irritation or even of inflammatory action after a protracted diarrhœa, is, one might suppose, an almost necessary consequence.* Is not the conjunctiva usually swollen and reddened after much weeping? and is not the urethra similarly affected when it has been irritated and inflamed? But it is unnecessary to pursue this subject, as it is not likely that any British physician will be biassed by such shallow analogies as our author has employed on the present occasion. What we chiefly regret is to see, that some of the very leading men in France, at the present day, are still so very far from the very threshold of a safe and

* Perhaps many of our readers will remember that not a few of the leading men in Paris, including *Dupuytren,* at the time of the invasion of the Asiatic cholera, came to the important pathological conclusion that the seat and proximate cause of this formidable stranger disease were an hypertrophied and (occasionally also) an ulcerated state of the Peyerian and Brunnerian glands of the intestines! Their favourite remedy, if we remember aright, was the acetate of lead—which, it was alleged, acted as a direct soothing and antiphlogistic sedative! The patients died, as a matter of course; the *post-mortem* was always most elaborately performed, and the appearances found on dissection were, we need scarcely add, in strict accordance with the doctrine in question!! So much for medical science in the 19th century.

truly scientific pathology. How can it be otherwise when they allow their minds to be so blinded and misled by preconceived opinions? The last paragraph in M. *Rayer's* remarks affords a striking example of this. He tells us that Veterinary writers have often observed in the bodies of animals, that have died of typhoid affections, a softened state of several of the viscera and various abnormal conditions of the blood; but that they have not noticed any lesion of the mucous glands of the intestines. The inference, that we should be inclined to draw from such a statement, is that the former phenomena are in fact the pathognomonic anatomical characters of the disease—a statement quite in accordance with what we find in the bodies of those who die of malignant fevers. M. *Rayer* however seems to take a different view of the case; for, without overlooking these phenomena altogether, he considers them as of very subordinate importance, in an ætiological point of view, to the altered condition of the mucous glands.—(*Rev.*)

---

### ON THE COINCIDENCE AND ANTAGONISM OF CERTAIN DISEASES.

We have already, in the present Number, made one or two allusions to this subject, which for some time past has been one of the favourite themes of discussion among the *Savans* of the French Academy. M. *Boudin*, (who was for some years with the army in Algeria and is now physician of the Military Hospital at Versailles), is one of the most active supporters of the new doctrine of antagonism of diseases,—so far at least as regards the mutual repulsion of marsh fevers on the one hand, and of typhus and pulmonary consumption on the other; and hence, as we are told, wherever the first are very prevalent, the latter are kept in abeyance, and *vice versâ*. This gentleman has written a long letter (apparently in reply to some strictures that had been made on his former observations) on the subject: from it we select the following somewhat *grandiloquent* extracts.

"Whenever a novel truth makes its appearance on the horizon of science, there arises almost immediately against it a host of opponents—men on whose banner cannot always be inscribed as a motto 'the love of truth.'

"If it be the destiny of every truth to require, like the fruits of the earth, a certain period of time before it can be matured, there are at the same time in man's heart two instinctive feelings which tend to secure the necessary proof or testing, so to speak, of this evolution. The first of these is the AMOUR-PROPRE of certain persons which leads them to deny every thing that is out of the usual course of received opinions; and the other is the *inertia* of the crowd, who are so apt, at once and without reflection, to accept the bold asseverations which an inexorable routine is ever ready to oppose to the progress of true knowledge. When a man of a certain amount of talent allows his mind to be impregnated with any error, it is rare that he does not succeed in environing it with a host of arguments, which, in the eyes of the multitude, constitute a satisfactory demonstration of its truth.

"It is little more than two months ago since a man, who holds a high place in the hierarchy of science (M. *Rayer*) suggested to the Academy to institute some enquiries as to the relative frequency of phthisis in marshy countries—a question mooted in my Treatise on Marsh Fevers, and subsequently developed at greater length in my Essay on Medical Geography.

"By adopting with acclamation this important suggestion, the Academy paid a solemn homage to the value of these opinions; but this very act was no sooner known than it became the signal for an outburst of violent opposition—which however has had no other effect but that of more firmly establishing the truth of the proposition in question.

"It was at first the Strasbourg school, that represented itself as refractory to the law of Antagonism. But it was no difficult task for me to demonstrate that the physicians there confounded the diseases of the marshy districts of Alsatia with those of the non-marshy districts; the diseases of the city, properly so called, of Strasbourg with those of its environs, including the citadel and other buildings; and lastly, the diseases that are truly and legitimately called endemic with those that are merely accidental or imported. I likewise pointed out the vague assertions and the utterly unsatisfactory and fallacious reasoning that were used by these gentlemen.

"The next adversary that presented himself was M. *Fourcault*, who pretended that I had far too much overlooked the agency of the mere moisture or dampness of places, as a potent cause in inducing various diseases. But if he had read with due attention what I have written on the subject, he must have seen that I have enumerated many facts to prove that the circumstance of humidity alone will not at all account for the immunity of certain places from phthisis and typhoid fever. At *Brest*, for example, which is damp but not marshy, we find that one out of every four of the convicts there die of pulmonary consumption; whereas at *Rochefort*, which is marshy as well as damp, the mortality from this disease does not exceed one in thirty-six.

"We are rejoiced to find that, with the view of investigating this subject, the Academy of Medicine at Athens has proposed as a subject of discussion at the approaching *Concours* the question, 'What is the amount of influence which the marshy districts of the country have on the development of tuberculous disease."

"Two centuries ago, intermittent fever was rife in London and its neighbourhood: James I. and Cromwell are reported to have died from it. Now-a-days the disease is scarcely at all known there; while phthisis and typhoid fever have unquestionably become greatly more frequent and destructive.

"Since the drying of an extensive marsh between the lakes of Zurich and Wallenstedt in Switzerland, ague has disappeared from the surrounding country: but, alas! consumption has become much more common in consequence. A similar pathological transformation is reported to have taken place in New York within the present century.

"To recur once more to Strasbourg, let us hear what M. *Forget* (who has very zealously impugned the new doctrine of antagonism) says of his practice, as it is from the data, obtained from this, that he deduces his main argument. 'At my clinique in the hospital, I have had 269 cases of typhoid fever and 230 of phthisis: the latter figure is indeed much below the actual number, as we do not admit any cases of pulmonary consumption into the wards, except when the disease is in its first stage.' Now even in this statement there is a confirmation of our doctrine that the two diseases, here named, are usually indigenous in one and the same place. M. *Forget* proceeds to say: 'In the course of these six years and four months, I have had 335 cases of intermittent fever, or very nearly one case during every week.' From this circumstance, the Professor infers that ague is very prevalent in Alsatia. We should have arrived at the very opposite conclusion. That there should be only one patient affected with intermittent fever, a week, in the hospital of a city, which contains 70,000 inhabitants, appears to us a pretty strong argument that the disease is of rare occurrence, and is certainly not endemic there. Besides, as we have already said, no person can judge of the diseases of the environs of Strasbourg, and of the surrounding country, by those of the city itself. We should also remember that the statistics of a single hospital are often apt to mislead us as to the prevailing diseases in any great town.

"In the military hospital at Marseilles, we very often find that the entire number of the cases of typhoid fever are supplied by one regiment of the garrison, while another regiment which may have arrived, it may be, from Corsica or Africa, shews itself quite refractory to this disease, and gives birth to those diseases only which have been endemic in the countries, where the troops have been residing. Here, then, we have an instance of a *hospital Coincidence,* but of a *geographical antagonism,* of different morbid conditions.

"At Strasbourg, on the other hand, there are never any arrivals from Corsica or Africa; but there is, as in all large towns, a floating population, whose former residence ought always to be well investigated by the philosophic physician, to enable him to treat their diseases with success. He must also not forget to take into his consideration the very important circumstance, that many of the inmates of its hospital are the inhabitants, not of the city itself, but of the surrounding country—whose prevailing diseases may be very different from each other. Here is an instance in point.

"The 60th regiment, which came from the Citadel of Strasbourg two months ago, has been obliged to leave a number of its men affected with ague along the whole line of its march; and we have still numerous cases sent to us at Versailles, while the prevailing disorders there are thoracic affections. Now if this regiment had sent its sick to the hospitals in Paris, we should then have had numerous examples of an alleged coincidence of ague with the usual diseases of the metropolis—in other words, we should have had an example of *hospital,* but not of *Endemic, Coincidence.*"—*Gazette Medicale.*

*Remarks.*—Like every other novelty in medical doctrine, that is brought forward in the present day, this alleged *law of Antagonism* between marsh fevers on the one hand, and Phthisis and Typhus on the other, will doubtless be pushed to an extravagant length for some time, and then it will be apt to be entirely neglected. In all ages, physicians must have observed that certain systemic diseases are rarely found to prevail simultaneously in any place. Influenza and genuine Typhus are seldom found to exist together; and it has often been noticed that the invasion of one exanthem suspends, or entirely arrests, the activity and prevalence of another. But this remark, it may be said, applies to *Epidemic* rather than to *Endemic* diseases. True; but most probably the law, that holds true in the one case, is equally applicable to the other.

That pulmonary consumption is not so prevalent in marshy agueish districts, as in those of a different formation, we are ready to admit to a certain extent; and perhaps for this very simple reason, that such localities are not exposed to those very vicissitudes of temperature which are, it is well known, the usually exciting cause of all chest complaints.

But can we say the same as to any antagonism or repulsion between Ague and Typhus fever?—it seems more than doubtful.

Are not the two diseases frequently blended, as it were, together in such places as New Orleans, Sierra Leone, and the banks of the Ganges? for what is the character of the destructive pestilences of these places but that of a malignant remittent fever—a disease intermediate between genuine continued and intermittent fevers, and partaking of the characters of both?

M. *Boudin* says that, since the disappearance of Ague in London, the frequency of Typhus and Phthisis have greatly increased in the metropolis; but then have we not good reason for believing, from the writings of Sydenham, that in his day grave continued fevers were quite as common, and indeed more so, than they are now? We suggest these circumstances, not so much with the view of denying the truth of M. *Boudin*'s doctrine, as in the hope of guarding him and others from carrying it to an extravagant and erroneous extent.—(*Rev.*)

### M. Raciborski on Purulent Infection.

"M. *Bonnet*, chief surgeon of the Hôtel Dieu at Lyons, has recently directed the attention of the profession to the good effects of cauterising—either with the actual or the potential cautery—ulcers and suppurating wounds, with the view of preventing or even of curing that very formidable affection which has been called purulent Phlebitis and purulent Infection. It appears to me that a temperate discussion of this question is calculated to throw some light on several topics in Pathology, that are a good deal canvassed in the present day.

"In my thesis, published in 1840, I examined with much care the various theories (of metastasis, absorption, aspiration, phlebitis, &c.) which have been proposed by different writers, to account for the formation of those internal abscesses or purulent deposits, and the occurrence of the other morbid affections, which not unfrequently accompany open wounds in a state of suppuration, and it then, as I stated at the time, seemed to me that none of them was satisfactory in an ætiological point of view. We can at present allude to the last only of these theories, and shall now very briefly state our reasons for refusing our assent to it.

"The doctrine of phlebitis reposes on the assumption that there is a passage of the pus—secreted by the inner surface of the veins in a state of inflammation, after operations, injuries, accouchement, &c.—into the current of the circulation. But it is very far from being proved that such a passage or transmission ever takes place. On the contrary, it would seem, from the researches of most pathologists in the present day, that the first phenomenon in actual phlebitis is the formation of a coagulum that plugs up the inflamed vessel, and which thus detaches the diseased from the healthy portion of the tube. In such a state of things, it is obvious that any purulent matter, which may be secreted in the former part, cannot become mixed with the general mass of the blood. M. *Dance* himself had felt the force of this objection so much, that, in order that he might be able to give some degree of consistency to his *ingenious romance*, (to use the expression of M. *Tessier*, to whom unquestionably belongs the merit of having first clearly established the importance of this point,) he was obliged to suppose that the secretion of pus takes place before the formation of the coagulum —a position at utter discordance with the observations of all the best pathologists. But, by a curious contradiction, when he was describing the symptoms of phlebitis, he enumerated in the last stage those which he considered to be owing to the passage of purulent matter into the blood. If space permitted, we might adduce other arguments that are unquestionably opposed to the doctrine that the symptoms, of what has of late years been called Purulent Infection of the system, are attributable to a suppurative inflammation of the veins."

"M. *Bonnet* seems to be one of those surgeons who attribute the constitutional disorders, which occasionally attend suppurating wounds, in part to an inflamed state of the veins, and in part, also, to the direct absorption of purulent matter into the system. But neither of these doctrines can well serve his purpose, when he attempts to give a reason for the successful results of cauterising the wounds themselves, or the adjacent veins, as a means of counteracting these disagreeable consequences.

"May not the following theory—derived from the study of *Liebig*'s beautiful researches—furnish a better explanation of the subject in question?

"It is well known to the chemist, that, if a small portion of yeast be placed in contact with a solution of sugar, the latter substance begins immediately to undergo certain changes, the result of which is the formation of alcohol and carbonic acid—and yet the yeast itself has lost nothing of its weight. May not something akin to this take place, when a portion of purulent matter, in a state of decomposition is brought into mere contact with the blood? and may not the entire mass of the circulating fluids in this manner undergo a certain change, as we daily see take place in the fermentation of large quantities of vegetable juices, when the minutest quantity of leaven is added to them?

"If we examine attentively the cases, in which the symptoms of *purulent infection*, or *purulent diathesis* appear, we shall find that these symptoms have almost always occurred under circumstances that are favourable to the decomposition of the secreted pus: indeed, the physical characters, as well as the chemical properties, of the purulent fluid abundantly prove the truth of this observation.

"The pus, having become vitiated—whether this has been from neglect of cleanliness in dressing the wound, or from the mere influence of an unwholesome atmosphere that may, perhaps, be charged with molecules in a state of putrefaction, or from the mere influence of an elevated temperature—communicates the movement of its decomposition to the coagulum which stops up the extremity, and thence to all the mass of the blood."

"It has usually been too much the case with pathologists to ascribe the alterations, which are met with in the veins of those who have died from suppurating wounds, to an inflammation of the parietes of those vessels. But I am persuaded that, in very many cases of this sort, the pus, that is found in the interior of the affected veins, is no more the direct consequence or result of a phlebitis, than is the deep red colour of their lining membrane, so frequently seen in fatal cases of typhus fever.

"In support of this opinion, I might allude to the accidents which are apt to follow dissection-wounds. In these cases all the phenomena, from the earliest local symptoms observed during life, to the most serious anatomical lesions discoverable after death, might seem to justify the appellation of Phlebitis,—which has been usually applied to this state;—and yet what is more manifest than that there is a primary alteration of the blood, under the influence of a fluid in the state of decomposition? Does not the success, which generally attends the immediate cauterisation of the wound in such cases, abundantly prove that it is to the destruction of the poisonous matter by the caustic employed, and not to the action of this on the parietes of the veins, that the curative effects are really attributable?

"It is in this manner that we should explain, I think, the cures obtained by M. *Bonnet* from cauterising wounds in a state of suppuration; and I am, at the same time, led to believe that, if we were to combine this practice with irrigations of cold water—so as to establish, as it were, a continual current of water around the solution of continuity—we might obtain most salutary results, and prevent many of those distressing and fatal accidents which too frequently supervene after severe accidents and operations."—*Archives de Medécine.*

---

### MISCELLANEOUS NOTICES.

### 1. *Notice of a new Danish Medical Journal.*

We have received the first number of the Bibliothek for Læger, edited by *Professor Otto* of Copenhagen, and published during the present year in that city. Its chief contents are—1. A paper on Phthisis and its treatment, with remarks on the use of Dr. *Ramadge's* inhaling apparatus. (The writer judges favourably of this mode of treatment! so much for his sagacity and judgment. We advise the doctor near Holborn to send a cargo of his little pipes forthwith to Copenhagen, addressed to the care of Dr. *Giersing*.) 2. Remarks on the most celebrated physicians of the French metropolis, and their characteristic methods of treatment. 3. A case of remarkable deformity of the male genital organs, by

Professor *Svitzer*. 4. On the advantage of silk ligatures by the same, &c., and also numerous clinical reports from the civil and military hospitals of Denmark. As we know nothing of the Danish language, we cannot do more than merely thank Professor Otto for his polite kindness in sending us the first number of his new periodical, and express our cordial good-wishes for its fame and success.

2. *Successor of Larrey in the Institute.*

The medical world in Paris had been, for some months before the day of election, in quite a buzz of expectation and *quidnunc-ery*, as to who should prove the fortunate candidate for the vacant place of the famous *Larrey*. The distinguished Professor of Montpelier, M. *Lallemand*, was the favourite, it was generally believed; and this idea was confirmed on finding that the committee, whose part it was to select a certain number of names out of the host of candidates, had placed him at the top of their list. The others followed thus: *Lisfranc, Ribes, Velpeau and Gerdy* (equal), *Amussat, Begin,* and *Jobert*. The indefatigable surgeon of La Charité, however, although only fourth on the list, proved at last to be the successful competitor. The French Journal, *l'Experience,* approves highly of the choice, and concludes its notice of the election with these words: "M. *Velpeau,* in point of professional fame, is one of the reigning princes of surgery. His works on operative surgery and topographic anatomy are known everywhere. He possesses in the highest degree a love for his art. His intellectual character is eminently progressive; if he errs in this respect, it is rather in the way of *too much* than of *too little.* His whole career has been one of labour and study; and no one has better proved by example the truth of the adage—

'Labor improbus omnia vincit.'"

(This *eloge,* we believe, is nothing more than true.)

M. *Lisfranc* does not fare so well. Some of the French journals are having a fling at him, for a very notable piece of puffery with which he is charged. It seems that the wealthy Marquis d'Aligre recently had the misfortune of breaking the neck of one of his thigh bones, and that M. *Lisfranc* was called in to attend him. This was all duly announced in the public journals, as a matter of course, at the time of the accident; and the profession naturally thought that there was an end of the matter, at least between the public and the doctor. Not so however; for we are told that many of the leading fashionables in Paris had the honour of receiving, some weeks after the accident, the following 'jolie petite lettre, fort coquettement lithographiée:'—

"The apparatus was yesterday removed from the limb of M. le Marquis d'*Aligre*. The fracture has healed, without any shortening of the member. From this time, the Marquis enters upon convalescence; he will soon be able to walk; his general health is excellent.

(Signed) *Lisfranc. Nauche.*"

Paris, 23d February, 1843.

3. *Transmission of Glanders by the Blood.*

M. *Renaut* recently exhibited to the Academy some morbid preparations illustrative of the effects of this disease, where it had been communicated by injecting into the veins of a healthy animal some of the blood taken from a glandered one. This fact however is not a new one; as *Blaine,* in his work on the veterinary art, expressly tells that when Mr. *Coleman,* on one occasion, bled an ass till it fainted, and then injected an equal quantity of blood drawn from the carotid artery of a horse affected with the glanders, the former animal exhibited in the course of a few days all the symptoms of the disease—a fact that was fully proved by the circumstance that the glanderous matter taken from it and inoculated upon another ass produced the usual effects of direct infection in this last.

*Vibourg* also, in his treatise on the Glanders and Farcy, has the following remark :—" Experiments have clearly shewn that the mass of the circulating fluid is really charged with the poisonous matter, and that, if glanderous blood is injected into the body of a healthy animal, the latter will become infected with the disease."

4. *Decadence of a brilliant Operation!*

How, in the name of all that is wonderful, comes it that we never hear now of dividing people's tongues—or, as we heard an elocution-professor once tell a public audience, of cutting people's throats—for the cure of stammering? This great achievement of modern surgery has lasted just about as long as a fashionable novel usually does: before a twelvemonth has expired, it is not even talked of. Last year the weekly periodicals teemed with cases of wonderful cures effected "slick off in a twinkling;" and now, alas! there is an utter dearth of any thing that excites curiosity. By mere accident we noticed the heading of an article in a late number of the Annales de la Chirurgie—by-the-bye, one of the best of the French journals—on the treatment of stammering by dividing the genio-glossi, and we were curious enough to see what the author would now say upon the defunct subject. With most praiseworthy candour, he tells his readers that, although he met with very decided success in some cases, the result of the operation in his practice, on the whole, was so unsatisfactory that for the last eight months he had entirely given it up. The editor of the journal, in a foot-note, remarks: "It is this conclusion alone that has induced us to insert the memoir of M. *Cloesser;* for it is more remarkable for its good faith, than for any importance of its statements."

5. *Iodine Injections in Hydrocele.*

The use of the tincture of iodine, more or less diluted, in cases of hydrocele as an injection, in lieu of wine, &c. is becoming more and more generally adopted by the French surgeons. M. *Velpeau* has recently written a long memoir, strongly recommending it; and we observe that M. *Pasquier*, surgeon of the Hôtel des Invalides in Paris, reports most favourably of its effects. It seldom produces much pain, even when the injection is strong; and, according to the experience of several surgeons, it seldom or never fails in producing the wished-for adhesive inflammation.

The action of the tincture of iodine on the skin appears to be very similar to that of a strong solution of the nitrate of silver; it stimulates the part for a short time, and then soothes irritation and pain. We have seen good effects from applying it daily to the integuments covering scrofulous and other indolent abscesses. Recently it has been recommended as very useful in strumous, and other kinds of, ophthalmia, when applied on the outside of the eyelids. It seems to act as a gentle counter-irritant or derivative, in the same manner as the nitrate of silver does.

6. *Affectation of would-be 'Savans.'*

A short time ago there was a long and unmeaning article by M. *Guerin*, in the Gazette Medicale, of which he is the 'redacteur en chef,' with this extraordinary heading—" of the scientific unity and solidarity (!) of anatomy, physiology, pathology and therapeutics in the study of the phenomena of the animal organism." We have rarely met with such a tissue of pompous absurdity—it possesses the rare union of German unintelligibility and French verbosity. As a matter of course, the 'refrain' of the whole is something about muscular retraction, deformities, and subcutaneous section of muscles, &c. A brick will suffice as a sample of the building: "The anatomy of ages has shewn me that, from early fœtal to adult life, the fibrosity of muscles in all animals alike goes on incessantly increasing, in relation to the fleshy constitution, that is to say in the ratio of the antiquity and the intensity of action of the cause." Reader, can'st thou tell us poor mortals what lingo this is; for we frankly confess that we do not understand one word of it? Surely this is the "darkening counsel by words without knowledge," that the wise Man of old hath so well exposed.

7. *Sulphate of Iron in Agues.*

In 1808, there was a great deal of intermittent fever in the low districts of Paris. The supply of cinchona in the metropolis at the time was nearly expended, and the drug was therefore to be had only at a very exorbitant price. This was owing to the circumstance that a most strict embargo had been laid by the French Emperor against every thing imported in British vessels from abroad; and it is well known that the French had scarcely a ship at sea. The Polish physicians had set the example of trusting chiefly to the use of the Arseniate of Soda; but this was found on experience to be a rather unsafe remedy to have recourse to in all cases. Dr. *Marc*—long one of the ornaments of French medicine—happily thought of the sulphate of iron, and used it in a great multitude of cases with the most decided success. Indeed in several instances it effected a cure of the disease, after bark had failed. M. *Corvisart*, the leading physician of the Court, wrote to Dr. *Marc* in the most flattering terms of compliment on the importance of his discovery. He received moreover a handsome reward from the Emperor.

We hear nothing in the present day of the use of Iron or indeed of almost any remedy save cinchona and its alkaloid basis, in the treatment of intermittent fevers. Perhaps this is to be regretted, as similar circumstances to those, in which M. *Marc* and his professional brethren were placed thirty years ago, may often recur to different nations. Some of the French physicians in Algeria report very highly of the substitution of arsenic for bark. One, for example, goes so far as to say:—

"It is the best febrifuge with which I am acquainted; for seventeen chronic cases, which had resisted the action of quinine and sulphate of iron, successively yielded to its employment. The average duration of the arsenical treatment was five days. I have seen a good many cases of the disease in Africa, in which bark and its various preparations failed in putting a stop to the morbid accessions.

"One great advantage of the arsenical solution is its tastelessness, and another, of even greater importance on many occasions, is its trifling expense. Every year France pays to South America considerably more than a million francs for the supply of cinchona; whereas the price of arsenic, as every one knows, is exceedingly small."

It is always well, in medical as well as in other matters, to have more than one string to our bow; and it is therefore much to be desired that the comparative efficacy of different anti-periodic medicines—either alone or in conjunction with bark—should be fairly tested on a large scale.

How strikingly does the action of those remedies, that have been found on experience to be really useful, shew that there is a marked analogy between Ague and Neuralgia.

8. *Droll Request of a learned Academician.*

"M. *Baudelocque* requests of the Academy that it will appoint a committee, before which he undertakes to prove that he never loses either a woman or an infant after delivery, whether this has been natural, or complicated with hæmorrhage, convulsions, præternatural position of the child, &c., *provided* he has been called in from the very commencement of the accident, and before any attempt has been made to terminate the accouchement. As a matter of course, he excepts from his proposal all cases of deformity of the pelvis, and of extra-uterine pregnancy."

Well, this modest request out-herods all the regular professional puffs we have ever met with. Hide your diminished heads, all ye Hygiests, Homœopathists,

Hydropathists, &c. &c. for most of you admit that you do meet with occasional failures; but here is a hospital physician tells us that he never does! These Frenchmen are surely strange mortals.

9. *Arsenic in Inveterate Syphilis.*

A woman, who had lost the whole of her palate from syphilitic ulceration, and was in a most deplorable state of suffering, so that there seemed scarcely any chance of ultimate recovery, was put upon a course of the Arsenical solution (*Fowler's*): she began with three drops, and gradually raised the daily dose up to thirty.

After continuing the use of the medicine until she had taken two ounces of the solution, the amendment was truly surprising; the ulceration was arrested, and the general health rapidly improved: the power of deglutition also was almost completely restored.

In the same German Journal (Hæser's Repertorium) is reported a well-marked case of scirrhus of the mamma, where the progress of the disease seemed to be quite arrested by the internal use of the Ioduret of Arsenic: in the course of six months, as much as 135 grains of this very active remedy were taken. It should be mentioned, however, that an issue was at the same time inserted in the corresponding arm—a remedy, by-the-bye, that should seldom be omitted in the treatment of all malignant formations.

(Of late, the Chlorate or Oxymuriate of Potass has been tried, by many of the hospital surgeons in this Metropolis, in cases of lupus, unhealthy ulcerations, &c. with very decided benefit—the dose from five to ten or twenty grains three times a day).

10. *The Use of Tea.*

A French Journalist thus learnedly writes:—"The difference in the vital instincts of different nations is remarkably exhibited by the use of this beverage (tea); for who, pray, are they which consume by far the greatest amount of it? Certainly neither the French, nor the Italians, nor yet the Spaniards; but the English and the Dutch—two nations that are perpetually immersed in a cold, heavy and damp atmosphere, and whose inhabitants are soft and flabby in flesh, and dull and phlegmatic in character. (What say you, John Bull, to your portrait?)

"The English and the Dutch cannot dispense with this, their favourite drink; and if they had not it, they would be obliged to have recourse to some other. (Very true; but what of that?) The use of tea certainly assists the powers of digestion, determines to the surface of the body, and proves a gentle stimulant to the whole system."

(Our wise neighbour writes as if the English and Dutch were the only people who make much use of tea. Not to mention the Americans, may we ask, do the Chinese, and all the Tartar tribes belong to the same category in point of national temperament? The would-be reasoning of the writer should, one might have thought, have led him to the very opposite conclusion; for if tea acts by being a stimulant, surely coffee is a much greater one; and therefore it should have suited a phlegmatic people best.)

11. *On the Utility of Moral and Physical Pain.*

Such is the title of a short work, recently published by M. *Mojon* of Geneva, and translated from the Italian by his friend Baron *Michel*. It is, says the reviewer in the Annales de la Chirurgie, a work as remarkable for the choice of its subject, as for the elevation of its sentiments, and the elegance of its language. The object of the author is to shew that pain is alike useful and necessary; and this he does, not only by most ingenious reasoning, but also by quoting the opinions of many distinguished philosopers in different ages. The testimony of

*Plato*, *Seneca*, *Cicero*, M. *Cousin*, not to mention that of many other authors, ancient as well as modern, is adduced and commented upon.

He cites numerous examples, too, from the records of history in support of his benevolent views. Altogether this little work is exceedingly well worthy a perusal, and is calculated to do good service to society.

(There is a beautiful chapter on this subject in the 'Student,' (a series of Essays) by Sir *E. Bulwer*. We remember being much pleased when we first read it; and, although that is now a good many years ago, we can recal to our mind several passages of it with no ordinary satisfaction. What concentration of thought there is in these two lines of our inimitable Shakespeare—

> " There is a soul of good in all things evil,
> Could men observingly distil it out."

Well would it be for the present age if our authors shewed that they *felt the force* of such a high-toned and masculine morality. Medical men, too, might often have it in their power to soothe the bodily and mental sufferings of the sick, far more effectually than they can do by stupefying the system with opiates, if they but availed themselves of every fit opportunity of administering the consolation that such a noble reflection is so well calculated to impart. The subject of Medical Ethics is not attended to, in the present day, nearly so much as it ought to be. We know not a finer theme for a work, that might be made both interesting and instructive, than this.)

### 12. *Entozoa in the Blood of Dogs.*

MM. *Gruby* and *Delafond* recently exhibited to the Academy the blood of a dog, in which numerous living *filariæ* were readily visible with the aid of a microscope. The size of these entozoa was about one-half or two-thirds of that of a blood-globule; their body was transparent and colourless; the anterior extremity was obtuse, while the posterior or caudal one terminated in a fine filament. The movement of these animals was very lively. When a drop of the blood was examined with the microscope, they were observed to be swimming about with a sort of undulatory motion among the globules, twisting and untwisting themselves with great vivacity. They continued alive for ten days after the blood had been drawn. They were found in the blood taken from all parts of the body, the jugular veins, the vessels of the abdomen, of the eye, &c.

The dog appeared to be in perfect health at the time. The writers say that this is the only instance in which they ever discovered the presence of these entozoary worms, although they have examined the blood of upwards of a hundred dogs at different times—some before, and others subsequently to, the present occasion.

### 13. *Extract of Cantharides, a useful Epispastic.*

M. *Soubeiran* has published, in a recent number of the Journal de Pharmacie et de Chemie, the formula for a blistering preparation, that is a good deal used in many parts of Germany. It is prepared by digesting with a gentle heat four parts of roughly powdered cantharides, one part of concentrated pyroligneous acid, and sixteen parts of alcohol, filtering the mixture, and slowly evaporating the fluid. The product has a buttery consistence; and, if smeared on a piece of paper, and thus applied to the skin, will be found to raise a blister in a short space of time. The consistence of this preparation, and more especially the presence of the acetic acid, suffice to prevent the cantharadine from crystallizing —a result which always takes place with the etherial extract, and constitutes a great objection to its use.

### 14. *Profanity of a French Medical Journal.*

While we denounce the ribald tone of some of the articles in the Examinateur

Medicale, conducted by MM. *Dechambre* and *Mercier*, we must do justice to the rest of the medical press in Paris, by acknowledging that we have never met with any thing in their pages calculated to give offence to the scruples of the most fastidious. In the Number of the Examinateur for the 1st of February last, the *feuilleton* article is headed, " the Anatomy, Physiology and Pathology of Man in another World." The writer, in a spirit of very profane levity, seeks to amuse his readers by quoting extracts from the Bible itself, but chiefly from the writings of some of the early Fathers of the Church, to shew that our bodies shall rise from the grave nearly in the condition in which they were when committed to the earth, with all their infirmities, and defects about them. We regret to say that many of the passages of the article are quite worthy of a countryman of *Voltaire*. The following is the closing paragraph; it is far less objectionable than much that precedes it; but it will suffice to convince our readers that we do not condemn unjustly. " In fine, it may be seen, by what we have said above, that all the elect will arise at the latter day in a state of perfect development, with their distinctive sex, and entirely exempt from all desires, diseases and deformities. And as it cannot for a moment enter into our thoughts that any of our dear *confreres*, to whom these lines are especially addressed, shall form part of the *bad grain*, this result of our pious investigations has filled our heart with an indescribable satisfaction; for, whatever other people may say, one is always well pleased to know ' *qu 'on fera bonne figure pendant l'eternité*.' "

As a *set-off* to this unfavourable specimen, we shall select a short passage from M. *Serres*' recent work on Transcendental Anatomy.

15. *The Harmony of Design throughout Creation.*

" Every thing is great, every thing is admirable in Nature; what may appear in it irregular and imperfect implies regularity and perfection; and order is maintained even in the midst of seeming disorder. But the human mind, accustomed to study the phenomena of life in the higher animals only, has formed to itself an absolute, and therefore very imperfect, idea of creation. Whatever does not attain to or passes beyond our conventional types, and whatever deviates from the most generally received arrangements, is often at once declared to be an infraction of the laws which we had supposed to exist, and, as such, it is sometimes rejected from the domain alike of Nature and of science. Hence has arisen a great deal of that ignorant presumption which has been displayed on the subjects of what have been called Organic Anomalies or Monstrosities, and which, at different times, has vainly attempted to restrict animal life and animal functions within certain limits that we imagined *ought to exist*.

We have seen that *Aristotle* fancied that the existence of a heart was essential to animal life. *Galen* bewildered himself amid the labyrinth of final causes, and boldly declared that many things were utterly impossible in Nature, merely because (the causes) were not apparent. Even *Haller*, in his old age, fell into the extravagance of admitting the existence of functions without the presence of organs; and it has been only within the last few years that organic anomalies have not been rejected with abhorrence from the field of physiology. All this appears to us very absurd *now;* and yet not a few physiologists, even in the present day, do not persist the less in the adoption of similar ideas. Thus it is that, because we think that we understand the plan on which the higher tribes of animals have been formed, we immediately declare that whatever deviates from this plan and is at variance with our established notions, must be at once rejected and placed in another category."

